# Reconstitution of Iterative Thioamidation in Closthioamide Biosynthesis Reveals Tailoring Strategy for Nonribosomal Peptide Backbones

**DOI:** 10.1002/anie.201905992

**Published:** 2019-08-07

**Authors:** Kyle L. Dunbar, Maria Dell, Evelyn M. Molloy, Florian Kloss, Christian Hertweck

**Affiliations:** ^1^ Dept. of Biomolecular Chemistry Leibniz Institute for Natural Product Research and Infection Biology, HKI Beutenbergstrasse 11a 07745 Jena Germany; ^2^ Transfer Group Antiinfectives Leibniz Institute for Natural Product Research and Infection Biology HKI Beutenbergstrasse 11a 07745 Jena Germany; ^3^ Chair of Natural Product Chemistry Friedrich Schiller University Jena 07743 Jena Germany

**Keywords:** antibiotics, biosynthesis, enzymes, natural products, thioamide

## Abstract

Thioamide‐containing nonribosomal peptides (NRPs) are exceedingly rare. Recently the biosynthetic gene cluster for the thioamidated NRP antibiotic closthioamide (CTA) was reported, however, the enzyme responsible for and the timing of thioamide formation remained enigmatic. Here, genome editing, biochemical assays, and mutational studies are used to demonstrate that an Fe‐S cluster containing member of the adenine nucleotide α‐hydrolase protein superfamily (CtaC) is responsible for sulfur incorporation during CTA biosynthesis. However, unlike all previously characterized members, CtaC functions in a thiotemplated manner. In addition to prompting a revision of the CTA biosynthetic pathway, the reconstitution of CtaC provides the first example of a NRP thioamide synthetase. Finally, CtaC is used as a bioinformatic handle to demonstrate that thioamidated NRP biosynthetic gene clusters are more widespread than previously appreciated.

Thioamides are formed through the oxygen‐by‐sulfur substitution of amide bonds, a seemingly minor change that has major repercussions for the physicochemical properties of the compound.^1^ Accordingly, site‐specific thioamidation has long been used by synthetic chemists to tune the properties of small molecules. In regard to natural products, only a modest number of thioamidated secondary metabolites are known.1c, 2 One notable example is closthioamide (CTA, Figure 1 A), a symmetric nonribosomal peptide (NRP) produced by the anaerobic bacterium *Ruminiclostridium cellulolyticum* DSM 5812.^3^ This DNA gyrase inhibitor harbors six thioamide linkages that are essential for its antimicrobial activity and is the only bacterial thioamide‐containing NRP identified to date.3a, 4

**Figure 1:**
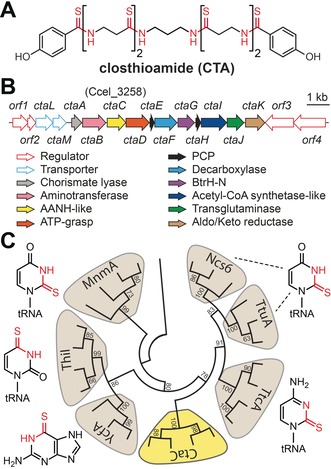
The CTA gene cluster contains an AANH homologue. A) Structure of CTA. B) The CTA biosynthetic gene cluster. AANH= adenine nucleotide α‐hydrolase, PCP=peptidyl carrier protein. C) Neighbor‐joining phylogenetic tree of AANH proteins (1000 bootstrap replicates). Structures of the compounds biosynthesized by each AANH subclass are shown with the installed moiety colored red. See the Supporting Information for a fully annotated cladogram.

Recently, we demonstrated that CTA is biosynthesized by an unusual thiotemplated, NRP synthetase (NRPS) independent pathway (Figure 1 B) and proposed a model for CTA maturation.^5^ However, the enzyme responsible for thioamide biosynthesis and the timing of thioamide formation remain unknown. Indeed, nothing is known about how these moieties are made in NRPs, in contrast to the great strides made towards understanding thioamide formation in both ribosomal peptides and nucleoside antimetabolites.1c, 6 Here, we identify and reconstitute the activity of a NRP thioamide synthetase (CtaC) involved in CTA maturation. Through phylogenetic analyses and biochemical assays, we demonstrate that CtaC is a novel member of the adenine nucleotide α‐hydrolase (AANH) protein superfamily that processes thiotemplated substrates. Through the use of substrate derivatives, we determine the timing of thioamidation and revise the CTA biosynthetic pathway. Finally, we leverage the sequence of CtaC to demonstrate that diverse anaerobic bacteria have the potential to produce thioamidated NRPs.

Of the genes present in the CTA biosynthetic gene cluster, we predicted that the product of *ctaC* (Ccel_3258) would be responsible for thioamide biosynthesis as it is a member of the AANH protein superfamily (Figure 1 C; see Figure S1 in the Supporting Information).^5^ Members of this superfamily catalyze transformations ranging from cofactor and antibiotic biosynthesis to the posttranslational modification of proteins.^7^ Although these reactions are varied, all involve ATP‐dependent adenylation of the substrate followed by nucleophilic attack at the activated position and elimination of AMP. Notably, some AANH proteins perform oxygen‐by‐sulfur substitutions on nucleotides in both primary and secondary metabolic pathways.6b, 7, 8 However, no AANH protein is known to thioamidate a peptidic substrate.

While analysis of the metabolite profile of CtaC‐deficient *R. cellulolyticum* indicated that CtaC is essential for CTA biosynthesis, no biosynthetic intermediates could be detected to clarify the role of this uncharacterized AANH protein.^5^ However, it is possible that the type II intron inserted in *ctaC* causes polar effects.^9^ We therefore used a CRISPR‐Cas9 genome editing method to generate a new *R. cellulolyticum ΔctaC* strain with a nonsense mutation in *ctaC* (see Figure S2).^5^, ^10^ As before, no oxygen isosteres of known CTA congeners were detected in culture extracts (see Figure S3). We next turned to an *Escherichia coli* heterologous expression system for the production of polythioamidated peptides.^5^ A vector containing all of the CTA biosynthetic genes except *ctaC* was constructed (pCTA‐*ΔctaC*; see Figure S4) and the metabolite profile of *E. coli* pCTA‐*ΔctaC* was checked for the accumulation of intermediates. Consistent with the *R. cellulolyticum* Δ*ctaC* results, no thioamide‐free intermediates were detected (see Figure S4).

To circumvent the difficulties in defining the role of CtaC by genetic approaches, we set out to characterize CtaC *in vitro*. Owing to solubility issues with His_6_‐tagged CtaC, the protein was isolated as an N‐terminal maltose binding protein (MBP) tagged fusion protein (see Figure S5). During isolation we noted that aerobically purified MBP‐CtaC solutions were slightly red, potentially indicating that CtaC binds iron. A recently identified tRNA‐modifying subfamily (TtcA/TtuA/Ncs6) of the AANH superfamily requires an iron‐sulfur cluster for activity.^11^ Indeed, CtaC has the [4Fe‐4S] cluster‐binding motif found in TtcA/TtuA proteins (see Figure S6).11a, 11c, 11d However, the UV‐visible spectrum of MBP‐CtaC had only a weak absorbance at the characteristic wavelength for such metal centers (ca. 410 nm,11a, 11c, 11d Figure 2 A) and purified with just 1.2±0.1 iron and 0.9±0.1 sulfide bound per protomer. To obtain MBP‐CtaC with an appropriately assembled iron‐sulfur cluster, the protein was treated with sulfide and ferrous iron in an anaerobic glovebox. The resultant MBP‐CtaC solution adopted a brownish‐red color, had an enhanced absorbance at 410 nm (Figure 2 A), and contained 4.5±0.2 iron and 4.5±0.1 sulfide per protomer.

**Figure 2:**
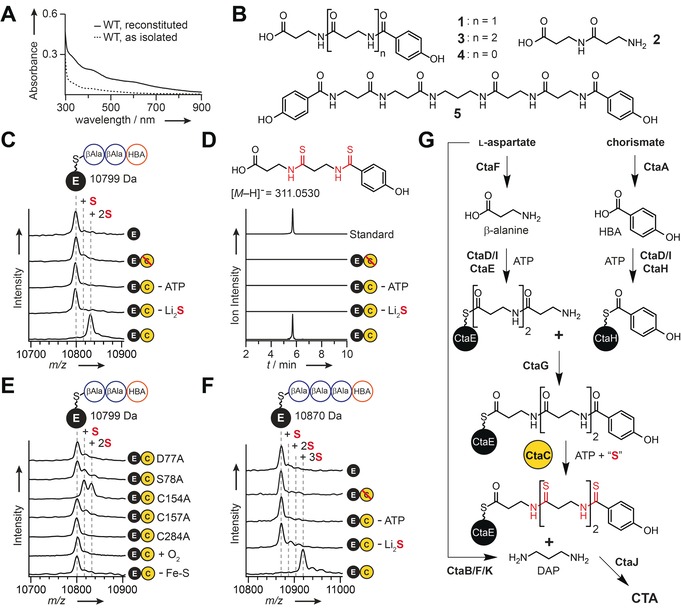
CtaC is a PCP‐dependent thioamide synthetase. A) UV‐visible absorbance spectra of as‐isolated and reconstituted MBP‐CtaC. B) Structures of substrates used in this study. C) Representative MALDI‐TOF‐MS spectral overlay of 6 h *in vitro* thioamidation reactions with **1**‐*holo*‐CtaE. D) LC‐HR‐MS profiles of thioamidation reactions with **1**‐*holo*‐CtaE following thioester cleavage. Traces correspond to the extracted ion chromatogram of the [*M*−H]^−^ ionic species for di‐thioamidated **1** and are displayed with *m*/*z* values ±5 ppm from the calculated exact mass. E) Representative MALDI‐TOF‐MS spectral overlay of 22 h *in vitro* thioamidation reactions with **1**‐*holo*‐CtaE and mutant CtaC enzymes, CtaC under aerobic conditions (+ O_2_), or *apo*‐CtaC (− Fe‐S). F) Representative MALDI‐TOF‐MS spectral overlay of 22 h thioamidation reactions with **3**‐*holo*‐CtaE. G) Updated biosynthetic scheme for CTA maturation. C, E, F) Dashed lines denote the expected shifts (+ 16 Da) obtained from the substitution of oxygen by sulfur. C, D, F) Red strikethrough=heat‐inactivated CtaC.

Based on the thiotemplated nature of CTA biosynthesis, we previously hypothesized that thioamide formation would occur on substrates linked to one of the two peptidyl carrier proteins (PCPs; CtaE/CtaH) encoded in the biosynthetic gene cluster (Figure 1 B).^5^ Thus, we purified CtaE and CtaH as N‐terminal His_6_‐tagged fusions and used matrix‐assisted laser desorption/ionization‐time‐of‐flight‐mass spectrometry (MALDI‐TOF‐MS) to verify that they were in their *apo* form (i.e. lacking the phosphopantetheinyl arm, see Figure S7). As the timing of thioamide formation during CTA maturation was unknown, we synthesized coenzyme A (CoA) conjugates differing by the presence or absence of 4‐hydroxybenzoic acid (HBA; **1**‐CoA and **2**‐CoA; Figure 2 B). These synthetic CoA‐linked substrates were loaded on the *apo*‐PCPs by the promiscuous phosphopantetheinyl transferase (PPTase) Sfp to generate the corresponding peptidyl‐*holo*‐PCPs (e.g. **1**‐*holo*‐CtaE; see Figure S7).^12^

With appropriately loaded PCPs prepared, we tested CtaC for thioamidation activity. As all AANH superfamily members require ATP for activity,^7^ thioamidation reactions were supplemented with ATP and magnesium. Lithium sulfide acted as the sulfur donor since a sulfur‐mobilization enzyme is not encoded in the CTA biosynthetic gene cluster and other sulfur‐inserting AANH enzymes have been shown to accept this non‐native sulfur donor *in vitro*.11b–11d, 13 All thioamidation reactions were performed in an anaerobic glovebox and progress was monitored by MALDI‐TOF‐MS. Although no processing of either **1**‐*holo*‐CtaH (see Figure S8) or **2**‐*holo*‐CtaE was observed (see Figure S9), the mass of **1**‐*holo*‐CtaE increased by approximately 32 Da compared to controls (Figure 2 C). This shift is consistent with the installation of two thioamides and was dependent on the presence of CtaC, ATP, and sulfide. To definitively identify the product of the CtaC reaction, the thioester linking **1** to *holo*‐CtaE was hydrolyzed, and the released products were analyzed by LC‐HR‐MS. A peak with a retention time and mass consistent with di‐thioamidated **1** was only observed in reactions containing all components (Figure 2 D; see Figure S10). Furthermore, MS/MS analysis of the product localized the sulfur atoms to the amide bonds (see Figure S10).

To probe the substrate scope of CtaC, thioamidation reactions were performed on substrates not linked to CtaE (**1**, **1**‐CoA, **3**‐CoA, **4**, and **5**; Figure 2 B). Despite using elevated enzyme concentrations and long reaction times, no turnover was observed by LC‐HR‐MS (see Figures S11–S15). Together, these data demonstrate that CtaC is a thiotemplated thioamide synthetase that functions on CtaE‐bound substrates and that sulfur insertion occurs after HBA is added to the poly‐β‐alanine backbone.

AANH enzymes adenylate their substrates and thus produce AMP as a byproduct of the reaction.^7^ When we measured the ATP hydrolysis products from thioamidation reactions with **1**‐*holo*‐CtaE by HPLC, both AMP and ADP were detected (see Figure S16). Moreover, neither the production of AMP nor ADP correlated with substrate processing by CtaC (see Figure S16). Although members of the AANH superfamily are highly divergent in both primary sequence and function, they share a fully conserved ATP‐binding motif that is used to present ATP for substrate adenylation.^7^ Thus, as an alternative approach to investigate the ATP‐dependent activity of CtaC, we generated mutant proteins bearing single alanine substitutions in the ATP‐binding pocket (CtaC_D77A_ and CtaC_S78A_; see Figures S5 and S6) and assayed the mutants for their ability to thioamidate **1**‐*holo*‐CtaE. As predicted, the activity of both mutants was abrogated (Figure 2 E; see Figure S17).

As mentioned above, members of the TtcA/TtuA family of sulfur‐inserting AANH enzymes require an oxygen‐labile [4Fe‐4S] cluster coordinated by three conserved cysteine residues for activity.11a, 11c, 11d Although the precise role is unclear, the prevailing hypothesis is that the cluster coordinates sulfide at the unique iron site and activates it for insertion into the substrate.^11^ To investigate the role of the Fe‐S cluster in thioamide formation by CtaC, we generated mutant proteins bearing single alanine substitutions to residues predicted to be involved in Fe‐S cluster‐binding (CtaC_C154A_, CtaC_C157A_, and CtaC_C284A_; see Figures S5 and S6). While all mutant proteins bound similar levels of iron and sulfide as wild‐type CtaC (see Figure S18), their thioamidation activity was diminished (Figure 2 E; see Figure S17). These results establish that although single mutations to the Fe‐S cluster binding site are not sufficient to fully disrupt cluster coordination, the activity of CtaC is highly sensitive to perturbations to its metal binding site. Notably, this phenomenon has been observed for other Fe‐S cluster dependent AANH enzymes.11a, 11d In further support of the essential nature of the metal center for CtaC activity, processing of **1**‐*holo*‐CtaE was completely lost in thioamidation reactions performed with either CtaC under aerobic conditions or with CtaC stripped of the Fe‐S cluster (Figure 2 E; see Figure S17).

Over the course of our studies we noted that regardless of the reaction time and concentration of CtaC used, tri‐thioamidated **1**‐*holo*‐CtaE was never detected (see Figure S19), accounting for only four of the six thioamides present in CTA. The initial biosynthetic model for CTA maturation posited that CtaC installs the final two thioamides after the dimerization of di‐thioamidated **1** through diaminopropane (DAP).^5^ However, the inability of CtaC to process free substrates casts doubt on this proposal. Indeed, products bearing three or four thioamidated β‐alanine residues were detected from *R. cellulolyticum* and the *E. coli* heterologous expression system, although their biosynthetic relevance was unknown.3b, 5 We therefore reasoned that a CtaE‐bound substrate containing three, rather than two, β‐alanine residues is the on‐pathway substrate for thioamidation by CtaC. Accordingly, **3**‐CoA (Figure 2 B) was synthesized, loaded onto CtaE *in vitro* using Sfp, and the conjugate (**3**‐*holo*‐CtaE) was tested for processing by CtaC. The MALDI‐TOF‐MS spectra demonstrated that the mass of **3**‐*holo*‐CtaE increased by approximately 48 Da compared to controls (Figure 2 F). Furthermore, using a combination of LC‐HR‐MS and LC‐HR‐MS/MS, the product was assigned as tri‐thioamidated **3** (see Figure S20).

In light of these data, we propose a revised biosynthetic pathway for CTA maturation (Figure 2 G). Briefly, the ATP‐grasp (CtaD) and acetyl‐CoA synthetase (CtaI) proteins are predicted to load β‐alanine and HBA onto CtaE and CtaH, respectively. HBA is synthesized from chorismate by CtaA^5^ and β‐alanine is either biosynthesized from aspartate by the decarboxylase CtaF, or taken from primary metabolism. The peptidyl transferase CtaG would transfer HBA from CtaH to tri‐β‐alanine‐CtaE to form **3**‐CtaE. Following the thioamidation of **3**‐CtaE by CtaC, DAP—which is potentially synthesized from aspartate by the sequential action of a reductase (CtaK), aminotransferase (CtaB), and decarboxylase (CtaF)—would be coupled to two molecules of tri‐thioamidated **3** by the transglutaminase CtaJ to form CTA.^5^ We suggest that the terminal β‐alanine remains on CtaE, thus serving as a priming residue that allows CtaC to concurrently install all thioamides in chemically equivalent positions (Figure 2 G).

As CtaC is the first example of a NRP thioamide synthetase, we sought to determine if this strategy for thioamidated NRP biosynthesis exists outside CTA maturation. To this end, we retrieved 100 homologues of CtaC from GenBank and generated a sequence similarity network with the protein sequences (Figure 3 A).^14^ Next, we surveyed the genomic neighborhood surrounding the *ctaC* homologue for genes encoding secondary metabolite biosynthetic enzymes (Figure 3 A). Notably, genes encoding thiotemplated biosynthetic enzymes were found in approximately 75 % of cases. In addition to a small number of CTA‐like biosynthetic gene clusters, many CtaC homologues are encoded in the vicinity of NRPSs (Figure 3 B), suggesting that this strategy for thioamidation is also used by canonical NRP biosynthetic pathways. Moreover, the majority of homologues were found in anaerobic bacteria (see Table S5), suggesting that anaerobes represent an untapped trove of thioamidated NRPs.

**Figure 3:**
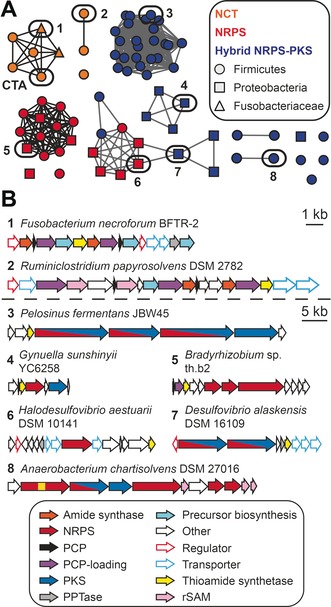
Homologues of CtaC are found in diverse thiotemplated biosynthetic pathways. A) Sequence similarity network of CtaC homologs. Nodes are colored based on the class of biosynthetic machinery found near the CtaC homologue, while their shape denotes the phylum of the organism harboring the gene cluster. NCT=non‐canonical thiotemplate, PKS=polyketide synthase. B) Representative biosynthetic gene clusters that encode a CtaC homolog (selected based on architecture diversity). Numbers correspond to the node numbering in panel A. rSAM=radical *S*‐adenosylmethionine enzyme.

In summary, we have reconstituted the first instance of thioamide formation in a NRP. The data presented herein demonstrate that this unusual thioamide synthetase functions on thiotemplated substrates and represents a new subfamily of the Fe‐S cluster dependent AANH enzymes. In addition to further clarifying the CTA biosynthetic pathway, the characterization CtaC serves as a foundation for the discovery of additional thioamidated NRPs. Future studies will focus on unearthing the native sulfur source for this enzyme and the characterization of the remaining steps in CTA maturation.

## Conflict of interest

The authors declare no conflict of interest.

## Supporting information

As a service to our authors and readers, this journal provides supporting information supplied by the authors. Such materials are peer reviewed and may be re‐organized for online delivery, but are not copy‐edited or typeset. Technical support issues arising from supporting information (other than missing files) should be addressed to the authors.

Supplementary
