# Supplementary material for: Reconstitution of Iterative Thioamidation in Closthioamide Biosynthesis Reveals Tailoring Strategy for Nonribosomal Peptide Backbones
Source: Angew Chem Int Ed Engl. 2019 Aug 7;58(37):13014–8. doi: 10.1002/anie.201905992 (PMC6772006; doi:10.1002/anie.201905992)
Supplement: Supplementary file 1 — Supplementary [file ANIE-58-13014-s001.pdf]

Angewandte Chemie
Eine Zeitschrift der Gesellschaft Deutscher Chemiker

Supporting Information

Reconstitution of Iterative Thioamidation in Closthioamide Biosynthesis Reveals Tailoring Strategy for Nonribosomal Peptide Backbones

*Kyle L. Dunbar, Maria Dell, Evelyn M. Molloy, Florian Kloss, and Christian Hertweck**

anie_201905992_sm_miscellaneous_information.pdf

Table of Contents

Experimental Procedures 2-12

Figure S1. Detailed phylogenetic tree of AANH enzymes 13

Figure S2. Generation of *Ruminiclostridium cellulolyticum* Δ*ctaC* by CRISPR-Cas9 genome editing 14

Figure S3. Amidated intermediates are not produced by *R. cellulolyticum* Δ*ctaC* 15

Figure S4. Amidated intermediates are not produced by *Escherichia coli* pCTA-Δ*ctaC* 16

Figure S5. SDS-PAGE analysis of purified proteins 17

Figure S6. Multiple sequence alignment of CtaC and TtuA/TtcA-type AANH enzymes 18

Figure S7. Phosphopantetheinylation of *apo*-CtaE and *apo*-CtaH *in vitro* by Sfp 19

Figure S8. Substrates presented on CtaH are not recognized by CtaC 20

Figure S9. CtaC does not recognize substrates lacking the HBA endcap 21

Figure S10. LC-HR-MS/MS analysis of thioamidated product from 1-*holo*-CtaE reaction 22

Figure S11. CtaC activity test with 1 23

Figure S12. CtaC activity test with 1-CoA 24

Figure S13. CtaC activity test with 3-CoA 25

Figure S14. CtaC activity test with 4 26

Figure S15. CtaC activity test with 5 27

Figure S16. Detection of ATP hydrolysis products from CtaC reactions 28

Figure S17. MALDI-TOF spectra of mutant CtaC reactions with 1-*holo*-CtaE 29

Figure S18. Fe-S cluster binding by CtaC mutants 30

Figure S19. Increased processing time does not lead to further processing of 1-*holo*-CtaE by CtaC 31

Figure S20. LC-HR-MS analysis of thioamidated product from 3-*holo*-CtaE reaction 32

Figure S21- S34. NMR spectra for synthetic compounds 33-39

Table S1. Strains used in this study 40

Table S2. Plasmids used in this study 41

Table S3. Oligonucleotide primers used in this study 42

Table S4. List of AANH proteins used to construct phylogenetic tree 43

Table S5. List of AANH proteins used to construct sequence similarity network 44-45

References 46

Experimental Procedures

General methods

Sequencing and oligonucleotide primer synthesis was performed by Eurofins Genomics. Media components were purchased from Sigma, Roth and Difco. All chemicals were purchased from commercial suppliers (Sigma, Roth, etc.) without further purification. Restriction endonucleases were purchased from New England Biolabs. A list of all strains, plasmids and oligonucleotide primers used can be found in **Table S1**, **Table S2**, and **Table S3**, respectively.

Bacterial strains and culturing conditions

Escherichia coli strains were grown in lysogeny broth (LB) shaken at 160 rpm or on LB agar plates at 37 °C with appropriate antibiotic selection (chloramphenicol, 25 µg mL^{-1}; kanamycin, 50 µg mL^{-1}; ampicillin, 100 µg mL^{-1}). All plasmid construction and storage was performed with *E. coli* TOP10, while *E. coli* Rosetta (DE3) and *E. coli* HM0079 were used for protein overexpression and production of CTA intermediates, respectively.

Ruminiclostridium cellulolyticum DSM 5812 was cultivated under an anaerobic atmosphere (N_2:H_2:CO_2, 85:5:10 vol:vol:vol) in a Whitley A35 anaerobic work station (Don Whitley Scientific) operating at 37 °C. Routine cultivation was performed in modified CM3 medium with cellobiose (6 g L^{-1}) as previously described.[1] For the production of CTA, strains were grown in DSMZ medium 165 as previously described.[2]

Bioinformatic analyses

For phylogenetic studies, the sequences were aligned with MAFFT 7[3] and the phylogenetic trees were reconstructed using the neighbor-joining method with 1000 bootstraps using MEGA6[4] (see **Table S4** for a list of sequences used). The PCP and AANH protein multiple sequence alignments were performed with Clustal Omega using the default parameters.[5]

Protein sequences for the sequence similarity network (SSN) were retrieved using BLASTp against the reference genome database and CtaC (Ccel_3258) as a query. The top 100 homologs of CtaC identified from this search (see **Table S5**) were used to generate the SSN using the Enzyme Function Initiative Enzyme Similarity Tool (EFI-EST; https://efi.igb.illinois.edu/efi-est/).[6] The network was constructed at an expectation-value (e-value) of 10^{-80} and visualized using Cytoscape (v. 3.2.1) with the organic layout.[7] Sequences with 100% identity were visualized as a single node in the network. The local genomic region (~10 kbp upstream and downstream) surrounding the gene encoding the CtaC homolog was checked for the presence of secondary metabolite biosynthetic genes. Nodes corresponding to proteins not found in a bioinformatically identifiable natural product biosynthetic gene cluster were removed from the final network; however, the accession numbers for these sequences can be found in **Table S5**.

LC-HR-MS

HPLC-HR-MS and HPLC-HR-MS/MS measurements were performed with a Thermo Accela HPLC-system coupled to either a QExactive Hybrid-Quadrupole-Orbitrap (Thermo Fischer Scientific) or Exactive Hybrid-Quadrupole-Orbitrap (Thermo Fischer Scientific) mass spectrometer equipped with an electrospray ion source. For QExactive measurements, separation was performed with an Accucore C18 column (2.1 × 100 mm, 2.6 µm, Thermo Fisher) operating at a flow rate of 200 µL min^{-1}, with 0.1% formic acid (solvent A) and acetonitrile + 0.1% formic acid (solvent B) and the following gradient: 5% solvent B for 1 min, 5% to 98% solvent B over 10 min, hold 98% solvent B for 12 min. For Exactive measurements, separation was performed with an Betasil C18 column (2.1 × 150 mm, 3 µm, Thermo Fisher) operating at a flow rate of 200 µL min^{-1}, with 0.1% formic acid (solvent A) and acetonitrile + 0.1% formic acid (solvent B) and the following gradient: 5% solvent B for 1 min, 5% to 30% solvent B over 1 min, 30% to 80% solvent B over 15 min, 80% to 99% solvent B over 1 min, hold 98% solvent B for 15 min.

MALDI-TOF-MS

Samples were acidified by the addition of trifluoroacetic acid (TFA) to a final concentration of 0.2% and desalted using a C18 ZipTip (Merck Millipore) according to the manufacturer's instructions. Protein mixtures were eluted from the ZipTip in 1.5 µL of 50% acetonitrile supplemented with 0.1% TFA. Eluates were diluted with 1.5 µL of 2% TFA and 1.5 µl 100 mM 2'-5'-dihydroxyacetophenone (DHAP) and spotted onto a MALDI plate. Samples were analyzed using a Bruker Daltonics UltrafleXtreme MALDI-TOF mass spectrometer. Spectra were obtained in linear positive mode and the instrument was calibrated to a commercially available standard (Protein Calibration Standard I, Bruker) prior to each measurement. Data analysis was performed with flexAnalysis 3.3 (Bruker).

NMR

1D-NMR (^{1}H, ^{13}C, DEPT) data were recorded in deuterated solvents (Deutero GmbH) on a Bruker AVANCE II 300 MHz or Bruker AVANCE III 600 MHz instrument equipped with a Bruker Cryo Platform. The chemical shifts are reported in ppm relative to the solvent residual peak (DMSO-d6: δ_H = 2.5 ppm, δ_C = 39.52 ppm; D_2O: δ_H = 4.79 ppm; MeOH-d4: δ_H = 3.31 ppm, δ_C = 49.15 ppm). For multiplicities of resonance signals, the following abbreviations are used: s = singlet, d = doublet, t = triplet, q = quartet, qt = quintet, br = broad.

General synthetic methods

The coupling reagents used in the following syntheses were 1-hydroxy-7-azabenzotriazole (HOAT), 1-hydroxybenzotriazole hydrate (HOBT), 1-ethyl-3-(3-dimethylaminopropyl)carbodiimide (EDCI) and (benzotriazol-1-yloxy)tripyrrolidinophosphoniumhexafluorophosphate (PyBOP). Boc-deprotection reactions were performed by the addition of a 95:5 mixture of TFA and water to the respective compound. Following a 15 to 30 minute reaction with stirring, the TFA was removed under nitrogen flow and the compound was dried under fine vacuum.

The following compounds were synthesized as previously described: closamide (**5**),[8] 3-(3-((tert-butoxycarbonyl)amino)propanamido) propanoic acid (**6**),[9] 3-(4-(tert-butoxy)benzamido)propanoic acid (**7**) and 3-(3-(4-(tert-butoxy)benzamido)propanamido) propanoic acid (**8**).[9]

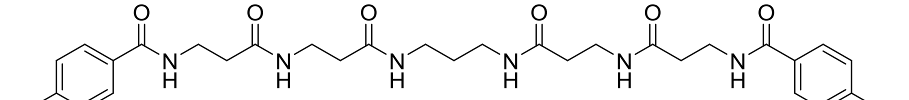

closamide, **5**

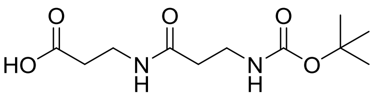

6

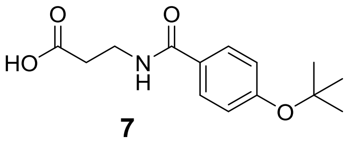

7

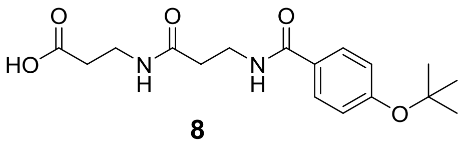

8

Synthesis of compound 1 [(3-(3-(4-hydroxybenzamido)propanamido)propanoic acid)]

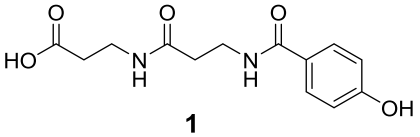

1

Compound **8** (compound 22 in Ref. 9) was deprotected using aqueous trifluoroacetic acid (95%) and concentrated under reduced pressure to obtain the title compound as a white powder (quantitative). See **Figure S21-S22** for NMR spectra.

^{1}H-NMR (300 MHz, DMSO-d_6): δ=12.11 (br s, 1H; OH), 9.90 (br s, 1H; OH), 8.19 (m, 1H; NH), 7.94 (m, 1H; NH), 7.67 (m, 2H; CH_2), 6.76 (m, 2H; CH_2), 3.39 (m, 2H; CH_2), 3.23 (m, 2H; CH_2), 2.28 – 2.38 ppm (m, 4H; CH_2); ^{13}C-NMR (75.5 MHz, DMSO-d_6): δ=172.9, 170.5, 165.9, 160.0, 129.0, 125.2, 114.7, 36.0, 35.4, 34.7, 33.9 ppm; HMRS (ESI, positive) *m/z* calculated for $C_{13}H_{17}N_2O_5$ $[M+H]^+$: 281.1132, found: 281.1132.

Synthesis of 1-CoA (HBA-di-β-alanine-CoA ester)

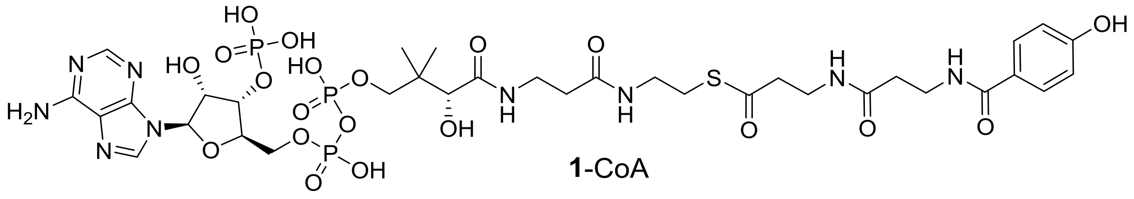

1-CoA

A solution of coenzyme A sodium salt (50.2 mg, 0.065 mmol, 1.1 eq.) in 500 µL water was added to a stirred solution of compound **8** (compound 22 in Ref. 9, 19.3 mg, 0.06 mmol), PyBOP (35.2 mg, 0.07 mmol, 1.2 eq.) and K_2CO_3 (24 mg, 0.17 mmol, 3 eq.) in 500 µL acetonitrile. The reaction was stirred for 2 hours and directly purified by preparative HPLC using a Shimadzu model (LC-8A pump system) HPLC equipped with a diode array detector (type: SPD-M20A) and a Phenomenex Synergi Hydro-RP 80 (250 x 21.2 mm, 10 µm) column to obtain the tBu-protected compound (22.2 mg, 36%). The mobile phase was composed of H_2O + 0.1% TFA (solvent A) and 83% acetonitrile (solvent B) and the following gradient was used for separation: 2% solvent B for 1 min, 2% to 10% solvent B over 4 min, hold at 10% solvent B for 5 min, 10% to 20% solvent B over 5 min, hold at 20% solvent B for 5 min, 20% to 40% solvent B over 5 min, hold at 40% solvent B for 5 min, 40% to 60% solvent B over 50 min, 60% to 100% solvent B over 3 min, hold at 100% solvent B for 2 min. The HPLC was operated at a flow rate of 20 ml min^{-1}. The purified compound was further treated with aqueous TFA (95%) to obtain **1**-CoA as a colourless powder (quantitative). See **Figure S23-S24** for NMR spectra.

^{1}H-NMR (600 MHz, D_2O): δ=8.46 (m, 1H; CH), 8.29 (d, ^3J(H,H)=20.7 Hz, 1H; CH), 7.48 (dd, ^3J(H,H)=8.4 Hz, ^4J(H,H)=33.3 Hz, 2H; CH), 6.74 (dd, ^3J(H,H)=8.4 Hz, ^4J(H,H)=37.6 Hz, 2H; CH), 6.20 (dd, ^3J(H,H)=5.4 Hz, ^4J(H,H)=18.4 Hz, 1H; CH), 6.07 (dd, ^3J(H,H)=6.1 Hz, ^4J(H,H)=23.0 Hz, 1H; CH), 4.50 (s, 1H; CH), 4.19 (m, 2H; CH_2), 3.89 (s, 1H; CH), 3.80 (dd, ^3J(H,H)=4.8 Hz, 1H; CH_2), 3.58 (dd, ^3J(H,H)=4.8 Hz, 1H; CH_2), 3.48 (dt, ^3J(H,H)=6.1 Hz, ^4J(H,H)=9.1 Hz, 2H; CH_2), 3.32 (m, 4H; CH_2), 3.06 (m, 2H; CH_2), 2.64 (m, 5H; CH_2), 2.40 (m, 3H, CH_2), 2.30 (q, ^3J(H,H)=6.3 Hz, 2H; CH_2), 0.82 (s, 3H; CH_3), 0.72 ppm (s, 2H; CH_3); ^{13}C-NMR (150 MHz, D_2O): δ=200.9, 174.5, 174.0, 173.7, 169.5, 162.6, 162.4, 159.2, 149.6, 148.2, 144.5, 142.3, 129.2, 124.7, 115.1, 87.5, 82.8, 74.1, 72.3, 69.7, 65.2, 42.5, 38.3, 36.3, 35.5, 35.4, 35.3, 35.0, 34.5, 27.9, 20.4, 18.3 ppm.

Synthesis of 2-CoA (di-β-alanine-CoA ester)

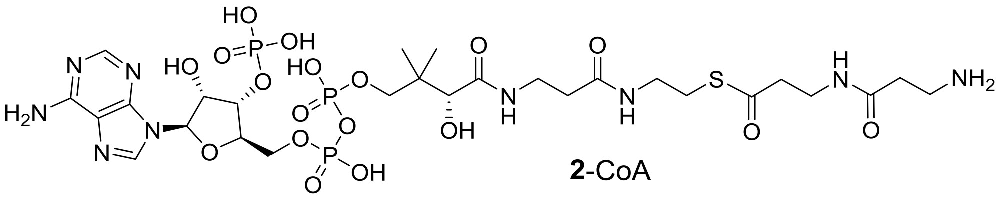

2-CoA

A solution of coenzyme A sodium salt (173.7 mg, 0.23 mmol, 1.2 eq.) in 500 µL water was added to 500 µL of compound **6** (compound 18 in Ref. 9, 50 mg, 0.19 mmol), PyBOB (114.4 mg, 0.22 mmol, 1.2 eq.) and K_2CO_3 (131.6 mg, 0.95 mmol, 5 eq.) in 500 µL acetonitrile while stirring. The reaction was stirred for 1.5 hours and directly purified by preparative HPLC using a Shimadzu model (LC-8A pump system) HPLC equipped with a diode array detector (type: SPD-M20A) and a Phenomenex Synergi Hydro-RP 80 (250 x 21.2 mm, 10 µm) column. The mobile phase was composed of H_2O + 0.1% TFA (solvent A) and 83% acetonitrile (solvent B) and the following gradient was used for separation: 2% solvent B for 1 min, 2% to 60% solvent B over 34 min, 60% to 100% solvent B over

5 min, hold at 100% solvent B for 5 min. The HPLC was operated at a flow rate of 18 mL min^{-1}. **2**-CoA was obtained after deprotection with aqueous trifluoroacetic acid (95%) in a yield of 65% as a white resin. See **Figure S25-S26** for NMR spectra.

^{1}H-NMR (300 MHz, D_2O/DMSO-d_6): δ=8.61 (s, 1H; CH), 8.40 (s, 1H; CH), 6.19 (d, ^{3}J(H,H)=5.2 Hz, 1H; CH), 4.57 (s, 1H; CH), 4.24 (s, 2H; CH_2), 3.98 (s, 1H; CH), 3.84 (dd, ^{3}J(H,H)=4.4 Hz, ^{3}J(H,H)=5.3 Hz, 1H; CH_2), 3.60 (dd, ^{3}J(H,H)=4.3 Hz, ^{3}J(H,H)=5.3 Hz, 1H; CH_2), 3.42 (dt, ^{3}J(H,H)=5.6 Hz, ^{3}J(H,H)=5.9 Hz, 6H; CH_2), 3.29 (m, 3H; CH_2), 3.19 (t, ^{3}J(H,H)=6.5 Hz, 4H; CH_2), 2.96 (t, ^{3}J(H,H)=6.1 Hz, 2H; CH_2), 2.80 (t, ^{3}J(H,H)=6.1, 2H; CH_2), 2.57 (t, ^{3}J(H,H)=6.4 Hz, 6H; CH_2), 2.40 (t, ^{3}J(H,H)=6.2 Hz, 2H; CH_2), 0.90 (s, 3H; CH_3), 0.79 ppm (s, 3H; CH_3); ^{13}C-NMR (75.5 MHz, D_2O/DMSO-d_6): δ=201.2, 176.1, 174.6, 174.0, 172.0, 163.0, 162.6, 144.6, 122.0, 118.1, 114.2, 110.4, 74.1, 72.1, 42.5, 38.4, 35.6, 35.5, 35.4, 35.2, 35.0, 33.2, 31.9, 31.8, 28.1, 20.7, 18.3 ppm.

Synthesis of 3-CoA (HBA-tri-β-alanine-CoA ester)

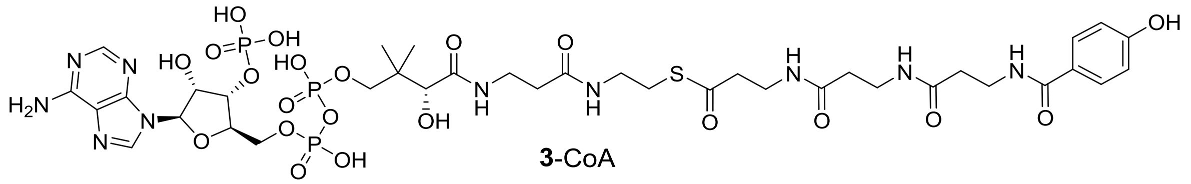

3-CoA

Compound **10** (16.5 mg, 0.04 mmol; see below) was mixed with PyBOP (29.4 mg, 0.06 mmol, 1.41 eq.) and K_2CO_3 (15 mg, 0.11 mmol, 2.7 eq.) in 500 µL acetonitrile. To this solution, coenzyme A sodium salt (36.3 mg, 0.05 mmol, 1.25 eq.) dissolved in 500 µL water was added and the reaction was stirred at room temperature for 1 hour. The mixture was directly purified by preparative HPLC using a Shimadzu model (LC-8A pump system) HPLC equipped with a diode array detector (type: SPD-M20A) and a Phenomenex Synergi Hydro-RP 80 (250 x 21.2 mm, 10 µm) column. The mobile phase was composed of H_2O + 0.1% TFA (solvent A) and 83% acetonitrile (solvent B) and the following gradient was used for separation: 2% solvent B for 1 min, 2% to 10% solvent B over 4 min, hold at 10% solvent B for 5 min, 10% to 20% solvent B over 5 min, hold at 20% solvent B for 5 min, 20% to 40% solvent B over 5 min, hold at 40% solvent B for 5 min, 40% to 60% solvent B over 50 min, 60% to 100% solvent B over 3 min, hold at 100% solvent B for 2 min. The HPLC was operated at a flow rate of 20 ml min^{-1}. The pure product was deprotected using TFA (95%) to obtain the title compound in a yield of 23% over two steps. See **Figure S27-S28** for NMR spectra.

^{1}H-NMR (600 MHz, D_2O): δ=8.57 (d, ^{3}J(H,H)=11.3 Hz, 1H; CH), 8.38 (d, ^{3}J(H,H)=20.7 Hz, 1H; CH), 7.59 (dd, ^{3}J(H,H)=30.5 Hz, 2H; CH), 6.86 (dd, ^{3}J(H,H)=37.4 Hz, 2H; CH), 6.18 (d, ^{3}J(H,H)=24.2 Hz, 1H; CH), 4.93 (dd, ^{3}J(H,H)=3.9 Hz, ^{3}J(H,H)=4.6 Hz, 1H; CH), 4.59 (s, 1H; CH), 4.87 (br, 1H; CH), 3.99 (s, 1H; CH), 4.17 – 4.33 (m, 2H; CH_2), 3.88 (dd, ^{3}J(H,H)=5.2 Hz, ^{3}J(H,H)=4.3 Hz, 1H; CH_2), 3.64 (dd, ^{3}J(H,H)=5.2 Hz, ^{3}J(H,H)=4.3 Hz, 1H; CH_2), 3.58 (dt (br), ^{3}J(H,H)=5.2 Hz, 2H; CH_2), 3.42 (t, ^{3}J(H,H)=6.6 Hz, 2H; CH_2), 3.38 (br, 2H; CH_2), 3.25 (t, ^{3}J(H,H)=7.1 Hz, 4H; CH_2), 3.24 (t, ^{3}J(H,H)=7.1 Hz, 2H; CH_2), 2.94 (dt, ^{3}J(H,H)=6.4 Hz, ^{3}J(H,H)=5.9 Hz, 2H; CH_2), 2.67 (dt, ^{3}J(H,H)=6.3 Hz, ^{3}J(H,H)=5.6 Hz, 2H; CH_2), 2.49 (t, ^{3}J(H,H)=5.9 Hz, 2H; CH_2), 2.40 (dt, ^{3}J(H,H)=6.6 Hz, ^{3}J(H,H)=6.3 Hz, 2H; CH_2), 2.34 (dt, ^{3}J(H,H)=6.3 Hz, ^{3}J(H,H)=5.8 Hz, 2H; CH_2), 0.92 (s, 3H; CH_3), 0.81 ppm (s, 3H; CH_3); ^{13}C-NMR (150 MHz, D_2O): δ=200.9, 174.6, 174.0, 173.9, 173.7, 169.7, 159.2, 149.7, 148.3, 144.5, 142.4, 129.2, 125.1, 118.5, 115.3, 87.5, 74.4, 74.1, 73.7, 72.2, 69.4, 65.1, 42.5, 38.5, 38.3, 36.4, 35.8, 35.6, 35.4, 35.2, 35.1, 34.9, 28.1, 20.7, 18.3 ppm.

Synthesis of compound 4 (3-(4-hydroxybenzamido)propanoic acid)

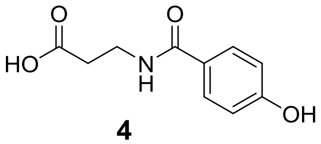

4

Compound **7** (compound 5f in Ref. 9) was deprotected using aqueous trifluoroacetic acid (95%) and concentrated under reduced pressure to obtain the title compound as a white powder (quantitative). See **Figure S29-S30** for NMR spectra.

^{1}H-NMR (300 MHz, DMSO-d$_6$): δ=12.12 (br s, 1H; OH), 9.91 (br s, 1H; OH), 8.23 (m, 1H; NH), 7.68 (m, 2H; CH), 6.77 (m, 2H; CH), 3.41 (m, 2H; CH$_2$), 2.47 ppm (t, ^{3}J(H,H)=7.0 Hz, 2H; CH$_2$); ^{13}C-NMR (75.5 MHz, DMSO-d$_6$): δ=172.9, 165.9, 160.0, 129.0, 125.1, 114.7, 35.4, 33.9 ppm; HMRS (ESI, positive) *m/z* calculated for $C_{10}H_{12}NO_4$ $[M+H]^+$: 210.0761, found: 210.0759.

Synthesis of compound 9 (Benzyl 3-(3-(3-(4-(tert-butoxy)benzamido)propanamido)propanamido)propanoate)

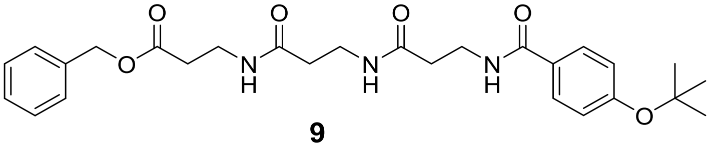

9

In order to obtain compound **9**, benzyl 3-aminopropanoate *p*-toluenesulfonate salt (91.5 mg, 0.26 mmol, 1.04 eq.) and compound **8** (compound 22 in Ref. 9, 84.6 mg, 0.25 mmol) were diluted in 20 mL DCM. The coupling reagent EDCI (59.5 mg, 0.31 mmol, 1.2 eq.) and Hünig's base (65 µL, 0.4 mmol, 1.5 eq.) were added to the mixture. The reaction was stirred for 19.5 h. The organic layer was washed with 20 mL 0.2 M NaOH, 20 mL 0.5 M HCl and 20 mL brine. The combined aqueous phases were extracted twice with 20 mL DCM and the combined organic extracts were dried over Na_2SO_4. The solvent was removed and the crude product was purified using a Shimadzu model (LC-8A pump system) HPLC equipped with a diode array detector (type: SPD-M20A) and a Phenomenex Luna C_{18} (10 µm, 250 x 21 mm) column. The mobile phase was composed of H_2O + 0.1% TFA (solvent A) and 83% acetonitrile (solvent B) and the following gradient was used for separation: 10% to 98% solvent B over 20 min, hold at 98% solvent B for 10 min. The HPLC was operated at a flow rate of 18 ml min^{-1}. Compound **9** was obtained in a yield of 50% as a colourless powder. See **Figure S31-32** for NMR spectra.

^{1}H-NMR (500 MHz, DMSO-d$_6$): δ=8.39 (t, ^{3}J(H,H)=5.6 Hz, 1H; NH), 8.02 (t, ^{3}J(H,H)=5.6 Hz, 1H; NH), 7.95 (t, ^{3}J(H,H)=5.6 Hz, 1H; NH), 7.76 (d, ^{3}J(H,H)=8.6 Hz, 2H; CH), 7.30 – 7.38 (m, 5H; CH), 7.02 (d, ^{3}J(H,H)=8.6 Hz, 2H; CH), 5.09 (s, 2H; CH$_2$), 3.43 (dt, ^{3}J(H,H)=6.7 Hz, ^{3}J(H,H)=6.3 Hz, 2H; CH$_2$), 3.29 (dt, ^{3}J(H,H)=6.6 Hz, ^{3}J(H,H)=6.1 Hz, 2H; CH$_2$), 3.23 (dt, ^{3}J(H,H)=6.8 Hz, ^{3}J(H,H)=6.2 Hz, 2H; CH$_2$), 2.51 (t, ^{3}J(H,H)=6.9 Hz, 2H; CH$_2$), 2.33 (t, ^{3}J(H,H)=7.2 Hz, 2H; CH$_2$), 2.21 (t, ^{3}J(H,H)=7.2 Hz, 2H; CH$_2$), 1.33 ppm (s, 9H; CH$_3$); ^{13}C-NMR (125 MHz, DMSO-d$_6$): δ=171.2, 170.5, 170.4, 165.7, 157.8, 136.1, 128.8, 128.4, 128.3, 128.0, 127.9, 122.3, 78.7, 65.5, 36.1, 35.4, 35.4, 35.3, 34.7, 33.8, 28.5 ppm; HMRS (ESI, positive) *m/z* calculated for $C_{27}H_{36}N_3O_6$ $[M+H]^+$: 498.2599, found: 498.2595.

Synthesis of compound 10 (3-(3-(3-(4-(tert-butoxy)benzamido)propanamido)propanamido)propanoic acid)

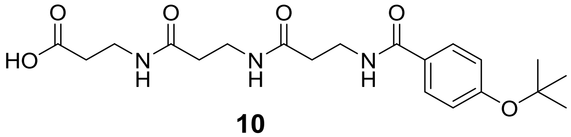

10

Compound **10** was obtained by benzyl deprotection of **9** (125.6 mg, 0.3 mmol). The reaction was performed in 10 mL MeOH using Pd/C (20.9 mg, 16% (w/w), 5% loading) while hydrogen was bubbled through the suspension in an ultrasonic bath for 6 hours. The product was used without further purification for the synthesis of **3**-CoA. See **Figure S33-34** for NMR spectra.

^{1}H-NMR (500 MHz, methanol-d$_4$): δ=7.59 (d, ^{3}J(H,H)=8.8 Hz, 2H; CH), 6.88 (d, ^{3}J(H,H)=8.9 Hz, 2H; CH), 3.46 (t, ^{3}J(H,H)=6.8 Hz, 2H; CH$_2$), 3.27 (t, ^{3}J(H,H)=6.7 Hz, 2H; CH$_2$), 3.24 (t, ^{3}J(H,H)=6.7 Hz, 2H; CH$_2$), 2.33 (dd, ^{3}J(H,H)=2.6 Hz, ^{3}J(H,H)=4.0 Hz, 4H; CH$_2$), 2.21 (t, ^{3}J(H,H)=6.7 Hz, 2H; CH$_2$), 1.23 ppm (s, 9H; CH$_3$); ^{13}C-NMR (125 MHz, methanol-d4): δ=175.4, 174.0, 173.8, 169.8, 160.2, 129.8, 129.5, 123.9, 80.3, 37.7, 37.1, 36.8, 36.6, 36.4, 34.6, 29.2 ppm; HMRS (ESI, positive) *m*/*z* calculated for $C_{20}H_{30}N_3O_6$ $[M+H]^+$ 408.2129, found: 408.2126.

Plasmid construction for CRISPR/Cas knockout vector

The knockout plasmid was generated as previously described with minor changes.[2] The identification of a suitable target site containing the necessary PAM (NGG) sequence for generation of the *ctaC* gene knockout was performed using the webtool CRISPy-web.[10] The sgRNA cassette (P4 promoter, sgRNA, spy terminator) containing the selected N20 sequence and the homology arms (250 bp upstream and downstream from the editing site with a mutated N20 sequence) for DNA repair following nicking by the Cas9-nickase were synthesized by GenScript. The resultant synthetic vector (pUC57-*ctaC*-target) was used as a template to amplify a DNA fragment with primers TargetCT-F/TargetCT-R (see **Table S3**) and Phusion High-Fidelity DNA polymerase (New England Biolabs) to create an amplicon including the N20-gRNA, the P4 synthetic promotor and the homologous regions with the mutated N20 sequence. Following purification using Monarch PCR and DNA Cleanup Kit (New England Biolabs), the amplicon was inserted into BsaI-digested pCasC[2] (New England Biolabs) using NEBuilder HiFi DNA Assembly Master Mix (New England Biolabs) to afford pCasC-*ctaC*. *E. coli* Top10 competent cells were transformed with the assembly reaction and transformants were selected on LB plates supplemented with 10 µg mL^{-1} gentamicin. Correct clones were identified by colony PCR using OneTaq Quick-load 2x Master Mix (New England Biolabs) using primers Cas-3258-check F /Cas-3258-check R, and verified by sequencing with Cas-3258-seq (see **Table S3**).

Generation of *Ruminiclostridium cellulolyticum* Δ*ctaC*

R. cellulolyticum Δ*ctaC*, which contains an in-frame nonsense mutation in *ctaC*, was generated using pCasC-*ctaC* as previously described.[2] The design of pCasC-*ctaC* was such that successful editing of *ctaC* would introduce the desired mutation (TAA), as well as an EcoRV restriction endonuclease site (GATATC) to facilitate screening of transformants. After electroporation, individual potential *R. cellulolyticum* Δ*ctaC* colonies were randomly picked and subjected to colony PCR using OneTaq DNA Polymerase (New England Biolabs) using primers Cas-3258-check F and Cas-3258-check R (see **Table S3**). The presence or absence of the restriction site (corresponding to edited or unedited Δ*ctaC*) was ascertained by digestion of the generated PCR products (1,276 bp) with restriction endonuclease EcoRV (New England Biolabs), followed by agarose gel electrophoresis (1.5% agarose gel) of the fragments. Pure mutant colonies were identified by the presence of the expected fragments of (851 bp and 425 bp) and lack of full-length PCR product (1,276 bp), while the PCR product remained undigested in the case of the wild-type control. Subsequently, genomic DNA was isolated from a putative mutant using a MasterPure Gram Positive DNA Purification Kit (Epicentre). In order to ensure the chosen mutant was free of wild-type contamination, the target gene was amplified and subjected to restriction analysis as described above. Purification of the undigested

Δ*ctaC* PCR product was performed using a Monarch PCR and DNA Cleanup Kit (New England Biolabs) and DNA sequencing (Eurofins Genomics Germany GmbH) using primer Cas-3258-seq (**Table S3**) was used to confirm the presence of the desired mutation (**Figure S2**).

Screen for production of CTA intermediates by *Ruminiclostridium cellulolyticum* Δ*ctaC*

Strains were cultivated and extracted with ethyl acetate as previously described.[2] Organic extracts were analyzed by LC-HR-MS (Exactive) for the presence of amide congeners of previously described intermediates.

Generation of pCTA1-Δ*ctaC*

For the generation of pCTA1-Δ*ctaC*, pCTA1 (see reference [2] for a description of the vector) was used as a template in a PCR amplification using the mutagenesis primers listed in **Table S3** and Q5 high-fidelity DNA polymerase (New England Biolabs). Template DNA was removed by digestion with DpnI (1 U; New England biolabs) for 4 h at 37 °C and the sample was purified using a Monarch PCR and DNA Cleanup Kit (New England Biolabs). The resultant linear PCR product (~70 ng) was treated with a mixture of T4 DNA polynucleotide kinase (5 U, New England Biolabs) and T4 DNA ligase (100 U, New England Biolabs) for 1 h at 25 °C to circularize the plasmid. The circularization reaction was used to transform *E. coli* Top10 cells and transformants were selected on LB agar supplemented 25 µg mL^{-1} chloramphenicol. Plasmids were isolated from the transformants and the absence of *ctaC* was verified by PCR using the primers listed in **Table S3** and by restriction digestion with EcoRI.

Screen for production of CTA intermediates by *Escherichia coli* pCTA-Δ*ctaC*

E. coli HM0079 pCTA2 competent cells were transformed with pCTA1-Δ*ctaC* and transformants were selected on LB plates supplemented with 100 µg mL^{-1} ampicillin and 25 µg mL^{-1} chloramphenicol. A single colony was used to inoculate 5 mL of LB supplemented with ampicillin/chloramphenicol and the culture was grown for 16-20 h at 37 °C and 160 rpm. The production of intermediates was performed in autoinduction medium according to the previously reported method[2] and in LB. For the LB experiments, 500 µL of this culture was used to inoculate 50 mL of antibiotic-supplemented LB in a sealed conical tube and cultures were grown at 30 °C and 130 rpm. When the cultures reached an optical density at 600 nm (OD_{600}) of approximately 0.6-0.8, they were supplemented with 50 µM $ZnCl_2$, 1 mM Na_2S, and protein expression was induced by the addition of 0.4 mM isopropyl-β-D-thiogalactopyranoside (IPTG). The induced cultures were grown at 30 °C and 130 rpm in sealed conical tubes for 3 hours. For both the autoinduction and LB cultures, the cells were separated from the spent medium by centrifugation at 4000 x *g* for 10 min.

Extraction of the cell-free spent medium was conducted as previous described[2] and extracts were checked for the presence of thioamide-free CTA precursors by LC-HR-MS (Exactive). Cell pellets were resuspended in 2.5 mL of 10 mM Tris pH 7.5 and lysed by sonication at 30% power for 2 min (SONOPLUS ultrasonic homogenizer with a MS73 microtip, Bandelin). In order to release any intermediates that might be bound to a PCP, KOH was added to the lysate to a final concentration of 10 mM and samples were incubated for 3 h at 37 °C. The base-treated lyasate was extracted with 2 volumes of ethyl acetate, the extract was dried over Na_2SO_4 and the ethyl acetate was removed using a rotary-evaporator. The resultant solid was dissolved in 100 µL of methanol and analyzed by LC-HR-MS (Exactive). See **Figure S4** for representative data.

Plasmid construction for *E. coli* expression vectors

The target genes (*ctaC*, *ctaE*, and *ctaH*) were amplified by PCR from the genomic DNA of *R. cellulolyticum* DSM 5812 using the oligonucleotide primers listed in **Table S3**. PCRs were performed with Phusion High-Fidelity DNA Polymerase (New England Biolabs) and amplicons were purified using an innuPREP Gel Extraction Kit (Analytik Jena). Following purification, the DNA fragments were either digested with NheI and BamHI (for pET28-*ctaE* and pET28-*ctaH*) or KpnI and BamHI (for pMAL-*ctaC*). The digested inserts were purified using an innuPREP PCRpure Kit (Analytik Jena) and ligated with an appropriately digested vector using T4 DNA ligase (New England Biolabs).

E. coli Top10 cells were transformed with the ligation reactions and transformants were selected on LB agar supplemented with appropriate antibiotic (pET28a, 50 µg mL^{-1} kanamycin; pMAL, 100 µg mL^{-1} ampicillin). Plasmids were isolated from the transformants and the sequence of the construct was confirmed by sequencing using the oligonucleotide primers listed in **Table S3**.

Oligonucleotide primers for the construction of pET28-*ctaC* were designed using the NEBuilder assembly tool (http://nebuilder.neb.com/) and are listed in **Table S3**. The gene was amplified from the genomic DNA of *R. cellulolyticum* DSM 5812 by PCR using Phusion high-fidelity DNA polymerase (New England Biolabs) and amplicons were purified using an innuPREP Gel Extraction Kit (Analytik Jena). The linear vector for the assembly reaction was generated by digestion of pET28a with NdeI and HindIII. The assembly reaction was performed using NEBuilder HiFi DNA Assembly Master Mix (New England Biolabs) according to the manufacturer's instructions. *E. coli* Top10 cells were transformed with the assembly reaction and transformants were selected on LB agar supplemented 50 µg mL^{-1} kanamycin. Plasmids were isolated from the transformants and the sequence of the construct was confirmed by sequencing using the oligonucleotide primers listed in **Table S3**.

Mutagenesis of pMAL-*ctaC*, pET28-*ctaE*, pET28-*ctaH* and pCTA1

Plasmids were amplified by PCR using the mutagenesis primers listed in **Table S3** and Phusion High-Fidelity DNA Polymerase (New England Biolabs). Template DNA was removed by digestion with DpnI (1 U; New England biolabs) for 4 h at 37 °C. Digested samples were used to transform *E. coli* Top10 cells and transformants were selected on LB agar supplemented with the appropriate antibiotic (100 µg mL^{-1} ampicillin, pMAL-*ctaC*; 50 µg mL^{-1} kanamycin, pET28-*ctaE/H*). Plasmids were isolated from transformants and verified by sequencing using the primers in **Table S3**.

Protein overexpression

E. coli Rosetta (DE3) cells were transformed with pET28a- and pMAL-derived expression vectors and transformants were selected on LB agar plates supplemented with 50 µg mL^{-1} kanamycin/25 µg mL^{-1} chloramphenicol and 100 µg mL^{-1} ampicillin/25 µg mL^{-1} chloramphenicol, respectively. A single colony was used to inoculate 4 mL of LB supplemented with the appropriate antibiotic and cultures were grown for 18-20 h at 37 °C and 160 rpm. The entire overnight culture was used to inoculate 400 mL of fresh LB medium supplemented with the appropriate antibiotic. Cultures were grown at 37 °C and 160 rpm until an optical density at 600 nm (OD_{600}) of approximately 0.6 was reached. Cultures were then iced for 10 min before protein expression was induced with the addition of IPTG to a final concentration of 0.4 mM. As CtaC is predicted to coordinate a structural zinc ion, CtaC overexpression cultures were supplemented with $ZnCl_2$ to a final concentration of 50 µM. Following induction, cultures were grown for an additional 16-18 h at 18 °C before the cells were harvested by centrifugation at 4000 x *g* for 15 min. Cell pellets were washed with Tris buffered saline (10 mM Tris pH 7.5, 150 mM NaCl) and stored at -20 °C for up to one month before use.

Purification of N-terminal His_6 fusion proteins

Cell pellets were resuspended in 30 mL of lysis buffer [50 mM Tris pH 8.0, 300 mM NaCl, 25 mM imidazole, 5% glycerol (v/v)] supplemented with 1 mg mL^{-1} lysozyme (Roth). After incubation on ice for 30 min, cells were disrupted by sonication at 4 °C using a SONOPLUS ultrasonic homogenizer with a MS73 microtip (Bandelin) and the following parameters: 30% power, three 45s cycles with 5 min breaks between cycles. The insoluble debris was removed from the lysate by centrifugation at 17000 x *g* for 30 min and the cleared lysate was loaded onto 2 mL of TALON Superflow resin (GE healthcare) equilibrated with lysis buffer. The resin was washed with 100 mL of lysis buffer supplemented with 1 mM dithiotheritol (DTT) and His_6-tagged proteins were eluted using 15 mL of elution buffer [50 mM Tris pH 8.0, 300 mM NaCl, 250 mM imidazole, 1 mM DTT, 5% glycerol (v/v)]. The eluent was concentrated in an appropriate molecular weight cutoff Amicon Ultra 15 mL centrifugal filter (Merck Millipore) and two 10-fold buffer exchanges with storage buffer [50 mM HEPES pH 7.5, 300 mM NaCl, 1 mM DTT, 20% glycerol

(v/v)] were performed in the filtration device prior to a final concentration step and storage at -80 °C. Protein concentration was determined by absorbance at 280 nm and purity was assessed by SDS-PAGE (**Figure S5**).

Purification of N-terminal MBP fusion proteins

Cell pellets were resuspended in 30 mL of lysis buffer [50 mM Tris pH 7.5, 500 mM NaCl, 5% glycerol (v/v)] supplemented with 1 mg mL^{-1} lysozyme (Roth) and lysed as described above. Following the removal of insoluble debris by centrifugation, the cleared lysate was loaded onto 2 mL of Amylose resin (New England Biolabs) equilibrated with lysis buffer. The resin was washed with 100 mL of lysis buffer supplemented with 1 mM dithiotheritol (DTT) and MBP-tagged proteins were eluted using 15 mL of elution buffer [50 mM Tris pH 7.5, 300 mM NaCl, 1 mM maltose, 1 mM DTT, 5% glycerol (v/v)]. The eluent was concentrated in an appropriate molecular weight cutoff Amicon Ultra 15 mL centrifugal filter (Merck Millipore) and two 10-fold buffer exchanges with storage buffer [50 mM HEPES pH 7.5, 300 mM NaCl, 1 mM DTT, 20% glycerol (v/v)] were performed in the filtration device prior to a final concentration step and storage at -80 °C. Protein concentration was determined by absorbance at 280 nm and purity was assessed by SDS-PAGE (**Figure S5**).

***In vitro* phosphopantetheinylation of the PCPs (CtaE/CtaH)**

Prior to phosphopantethein loading, the His_6-tags were removed from the *apo*-PCPs by the addition of thrombin (Novagen; final concentration of 0.5 U μL^{-1}) to a 30 µM solution of the PCP in reaction buffer [50 mM HEPES pH 7.5, 150 mM NaCl, 20 mM $MgCl_2$, 1 mM Tris(2-carboxyethyl)phosphine (TCEP)]. Following incubation at 30 °C for 2 h, PCP loading was initiated by the addition of 0.5 µM Sfp PPTase (New England Biolabs) and either 100 µM coenzyme A (Sigma) or 100 µM synthetic coenzyme A ester (**1**-CoA, **2**-CoA or **3**-CoA). PCP loading was allowed to proceed for 1 h at 30 °C before the samples were analyzed by MALDI-TOF-MS.

In order to generate *holo*-PCPs loaded with potential CtaC substrates, thrombin cleavage and Sfp PPTase loading reactions were performed under strictly anaerobic conditions [atmosphere composed of (N_2:H_2:CO_2, 85:5:10 vol:vol:vol)] in a Coy Lab anaerobic chamber at 25 °C. Following PCP loading, the *holo*-PCPs were purified using a PD-10 desalting column (GE Healthcare) equilibrated with anaerobic reaction buffer. PCP-containing fractions were pooled and concentrated using a 3 kDa Amicon Ultra 0.5 mL centrifugal filter (Merck Millipore) in the anaerobic chamber. Following concentration, glycerol was added to a final concentration of 20%, the protein solution was aliquoted into air-tight microfuge tubes and stored at -80 °C until use. The concentration of the loaded PCP was determined by absorbance at 280 nm and the BCA method (Pierce BCA kit, Thermo Fisher Scientific).

CtaC Fe-S cluster reconstitution

The reconstitution of the iron-sulfur cluster in MBP-tagged CtaC was performed under anaerobic conditions in a Coy anaerobic chamber. First, 25 µM MBP-CtaC in reconstitution buffer [50 mM HEPES pH 7.5, 150 mM NaCl, 2 mM $MgCl_2$, 1 mM TCEP] was mixed with 2.5 mM sodium dithionite and incubated at 8 °C for 1 h. To this mixture, ammonium iron citrate was added slowly to a final concentration of 250 µM with careful mixing. After incubation at 8 °C for 5 min, Li_2S was added to a final concentration of 250 µM and the mixture was incubated at 8 °C for 18 h. Unbound iron and sulfide was removed using a PD-10 desalting column (GE Healthcare) equilibrated with anaerobic reconstitution buffer. CtaC-containing fractions were pooled and concentrated using a 10 kDa Amicon Ultra 0.5 mL centrifugal filter (Merck Millipore) in the anaerobic chamber. Following concentration, glycerol was added to a final concentration of 20%, the protein solution was aliquoted into air-tight microfuge tubes and stored at -80 °C until use.

Chemical quantification of CtaC iron and sulfur content

The iron content of CtaC was quantified using a modified version of the previously established Fe^{2+}-ferene complex assay.[11] First, 10 µL of MBP-CtaC (25 µM) was diluted to 20 µL with distilled water. Iron was released from the protein by the addition of 20 µL of 1% HCl and heating to 80 °C for 10 min. After cooling the sample to room temperature, 100 µL of ammonium acetate [7.5% (w/v)], 20 µL of ascorbic acid [4% (w/v)], 20 µL of sodium

dodecylsulfate [2.5% (w/v)] and 20 µL of ferene [1.5% (w/v)] were added. Samples were mixed by vortexing, 100 µL was transferred to a 384-well microtiter plate and the absorbance at 593 nm was measured using a Varioskan Lux microplate reader (Thermo Fisher scientific). The iron content was determined by comparison to a standard curve generated with 25–400 µM $FeSO_4$. All measurements were performed in triplicate.

The sulfide content of CtaC was quantified using a modified version of the previously established methylene blue assay.[11-12] First, 10 µL of MBP-CtaC (25 µM) was diluted to 40 µL with distilled water. Following the addition of 120 µL of zinc acetate [1% (w/v)] and 10 µL of NaOH [7% (w/v)], samples were incubated at 22 °C for 15 min. The samples were centrifuged at 3000 x *g* for 30 s before the addition of 30 µL of N,N'-dimethylbenzene-1,4-diamine [0.1% (w/v), in 5 M HCl] and 30 µL $FeCl_3$ [10 mM, in 1 M HCl]. The pellet was resuspended by vortexing briefly, the color was allowed to develop for 20 min at 22 °C, 100 µL was transferred to a 384-well microtiter plate and the absorbance at 670 nm was measured using a Varioskan Lux microplate reader (Thermo Fisher scientific). The sulfide content was determined by comparison to a standard curve generated with freshly made 12.5–400 µM Li_2S. All measurements were performed in triplicate.

UV-visible Spectroscopy

Spectra were measured using a Varioskan Lux microplate reader (Thermo Fisher scientific) plate reader and a 384-well microtiter plate with a 50 µL sample volume. All protein solutions were analyzed at a concentration of 25 µM. Glycerol-containing reconstitution buffer was used as a blank.

CtaC activity assays

Assays with PCP-bound substrates were performed with 30 µM of the indicated PCP and 0.5 µM MBP-CtaC in reaction buffer [50 mM HEPES pH 7.5, 150 mM NaCl, 20 mM $MgCl_2$, 1 mM TCEP] supplemented with 2 mM ATP under strictly anaerobic conditions in a Coy anaerobic chamber. Lithium sulfide was used as a sulfur source for the reaction at a final concentration of 4 mM. Reactions were allowed to proceed for 6 h or 22 h at 25 °C before processing was measured by either MALDI-TOF-MS or LC-HR-MS (following cleavage of the substrate from the PCP; see below). Experiments to test the effect of oxygen on CtaC were performed outside of the anaerobic chamber with aerobic solutions. In experiments using *apo*-MBP-CtaC, the metal center was removed from the enzyme by treating a 50 µM protein solution with 10 mM EDTA for 3 h at 25 °C. EDTA was removed using a PD-10 desalting column (GE Healthcare) equilibrated with anaerobic reconstitution buffer. CtaC-containing fractions were pooled and concentrated using a 10 kDa Amicon Ultra 0.5 mL centrifugal filter (Merck Millipore) in the anaerobic chamber. Control reactions were either performed with heat-inactivated MBP-CtaC (incubation at 99 °C for 10 min) or by omitting MBP-CtaC from the reactions.

Assays with non-PCP bound substrates (free substrates) were performed with 0.5 mM of the indicated substrate, 2 mM ATP, 4 mM Li_2S and either 0.5 µM MBP-CtaC (reactions with **1**-CoA and **3**-CoA) or 1 µM MBP-CtaC (reactions with **1**, **4**, and **5**). Additionally, reactions with **5** were performed with 5 µM MBP-CtaC. All reactions were performed under strictly anaerobic conditions and were allowed to proceed for 22 h at 25 °C before product formation was monitored using LC-HR-MS (QExactive).

LC-HR-MS detection of PCP-bound thioamidated products from CtaC reactions

Potassium hydroxide was added to reaction mixtures to a final concentration of 10 mM and samples were incubated at 30 °C for 1 h. Next, one volume of methanol was added to the sample, precipitate was removed by passing the sample through a 0.45 µm syringe filter and substrate processing was determined by LC-HR-MS (QExactive). Products were compared to authentic thioamidated CTA congeners in the organic extract of *R. cellulolyticum* cultures.

Detection of ATP-hydrolysis products

Reactions were performed with 0.5 µM CtaC and 30 µM **1**-*holo*-CtaE, 1 mM ATP [50 mM HEPES (pH 7.5), 150 mM NaCl, 20 mM $MgCl_2$, 1 mM TCEP] for 18 h at 25 °C in an anaerobic chamber. The reactions were stopped by the addition of 1 volume of methanol and analyzed by HPLC according to an established method.[13] The HPLC profiles were compared to those of reference compounds (Jena Bioscience).

Figure S1. Detailed phylogenetic tree of AANH enzymes. Neighbor-joining phylogenetic tree of diverse sulfur-inserting AANH superfamily members with 1000 bootstrap replicates. The structures of the products synthesized by each AANH subclass are displayed with the installed moiety colored red. See **Table S4** for a list of the sequences used to generate this tree. GMP synthetase is used as an outgroup to root the cladogram.

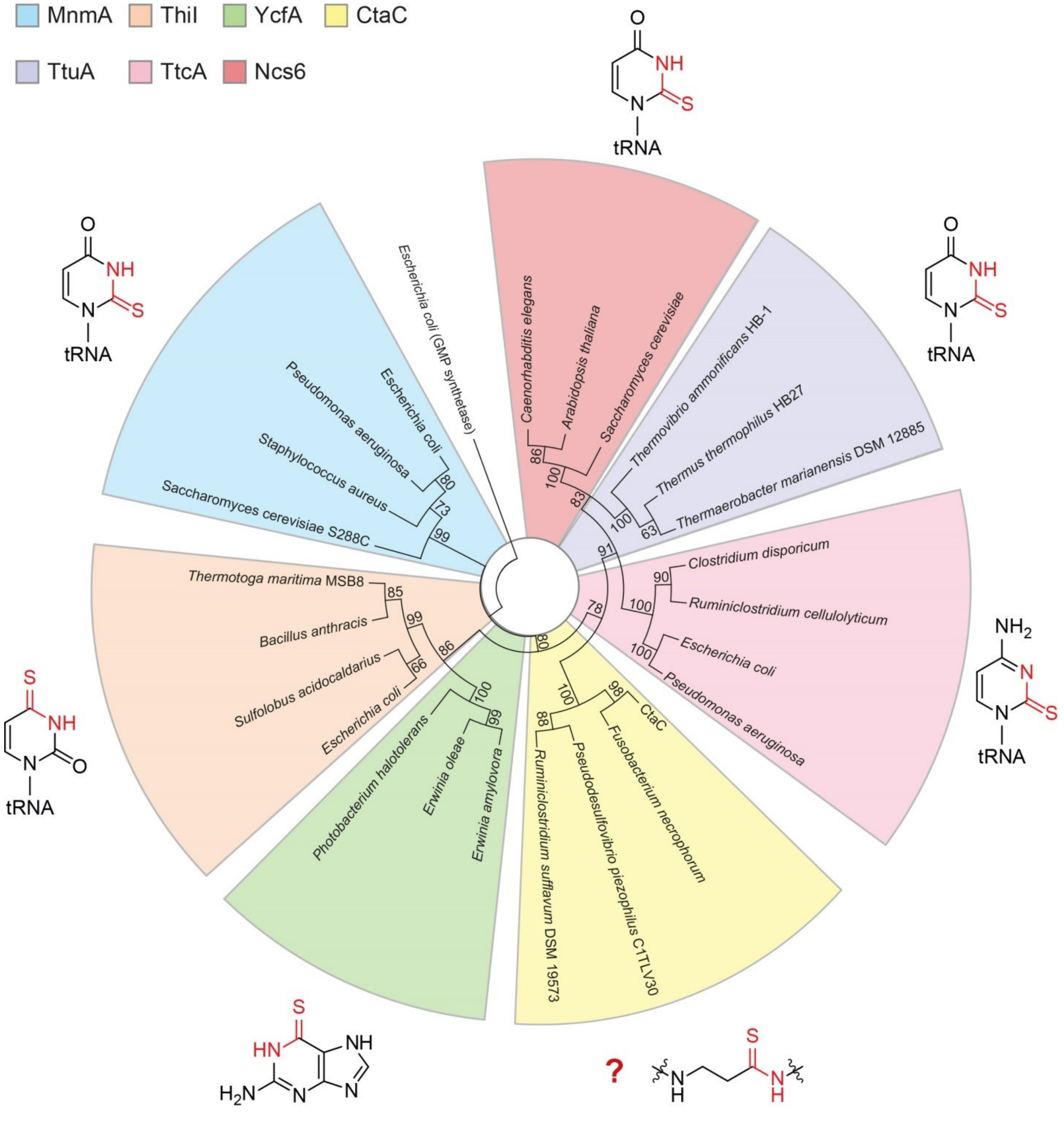

Figure S2. Generation of *Ruminiclostridium cellulolyticum* Δ*ctaC* by CRISPR-Cas9 genome editing. (A) Scheme depicting the CRISPR-Cas9 genome editing method used for the incorporation of a nonsense mutation and an EcoRV recognition site into *ctaC*. **(B)** Agarose gel (1.5%) of EcoRV-digested *ctaC*-containing PCR products amplified from gDNA isolated from the indicated strains. While the PCR amplicon from the wild-type control does not contain an EcoRV recognition site and is not digested by EcoRV, the amplicon from the Δ*ctaC* strain is digested to the expected 851 bp and 425 bp bands in the presence of EcoRV. L, GeneRuler 1 kb DNA ladder (Thermo Scientific). **(C)** The sequence of Δ*ctaC* in the region of the targeted mutation (colored red) is displayed beneath the corresponding sequence of *ctaC*. The location of the stop codon is indicated by an asterisk. **(D)** DNA sequence chromatogram verifying the precise insertion of the mutated sequence (highlighted) in *R. cellulolyticum* Δ*ctaC.*

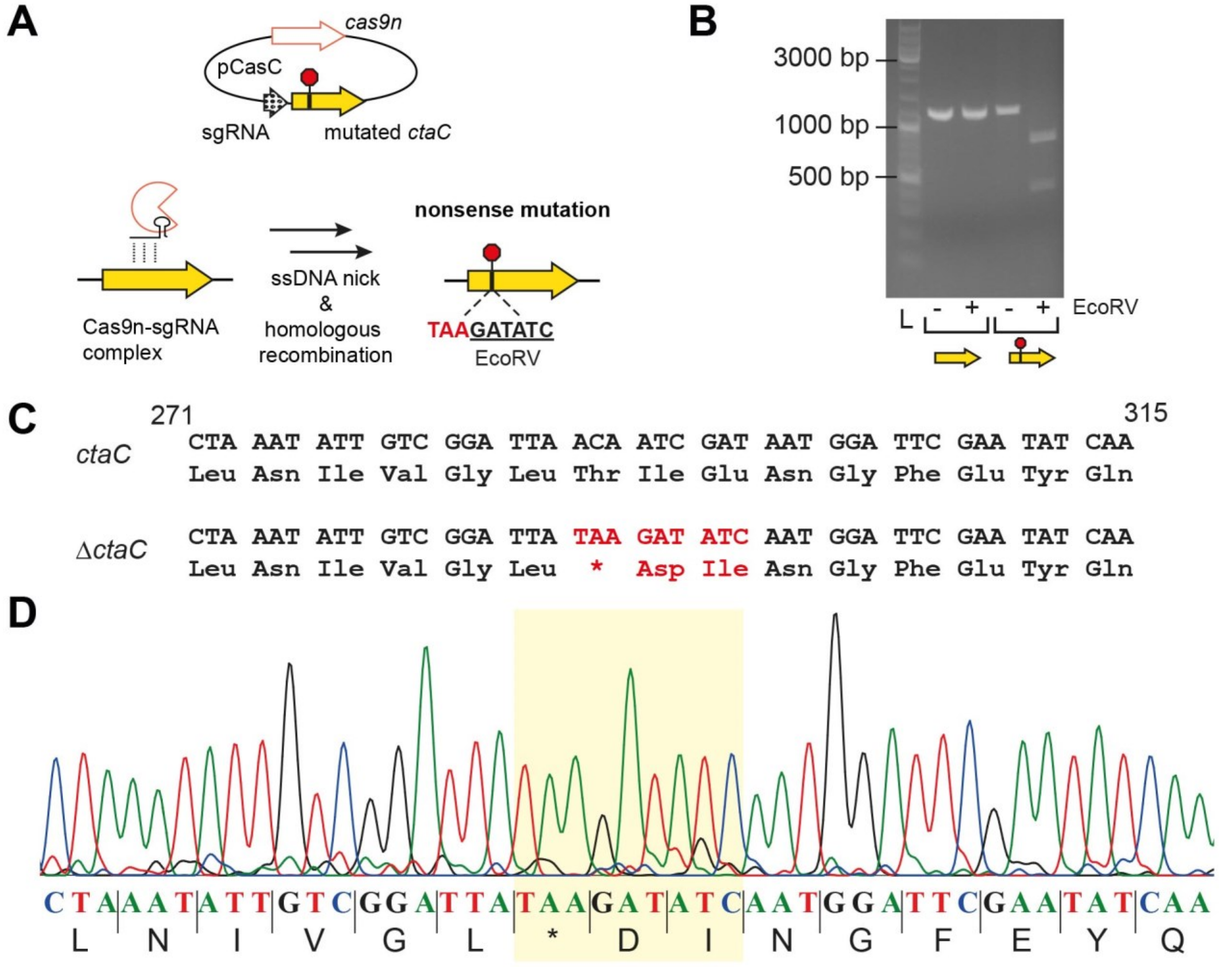

Figure S3. Amidated intermediates are not produced by *R. cellulolyticum* Δ*ctaC*. (A) Structures of known CTA congeners[2, 14] along with their corresponding oxygen isosteres. The expected *m/z* value for the [M-H]- ionic species is displayed for the oxygen isosteres. **(B, C)** LC-HR-MS profiles for organic extracts of *R. cellulolyticum* wild-type (WT) and *R. cellulolyticum* Δ*ctaC* cultures. Peaks corresponding to the CTA congeners shown in panel A are labeled in the UV-Vis profile. Asterisks denote peaks that are not related to CTA biosynthesis that are visible in the UV-vis profile of the mutant strain. Hash symbols correspond to breakdown products of CTA formed from the exchange of sulfur with oxygen. EICs corresponding to the [M-H]- ionic species for closamide and oxygen isosteres of the CTA congeners shown in panel A are displayed below the corresponding UV-vis profile. All EICs are displayed with *m/z* values ± 5 ppm from the calculated exact mass for the compound. HBA, 4-hydroxybenzoic acid.

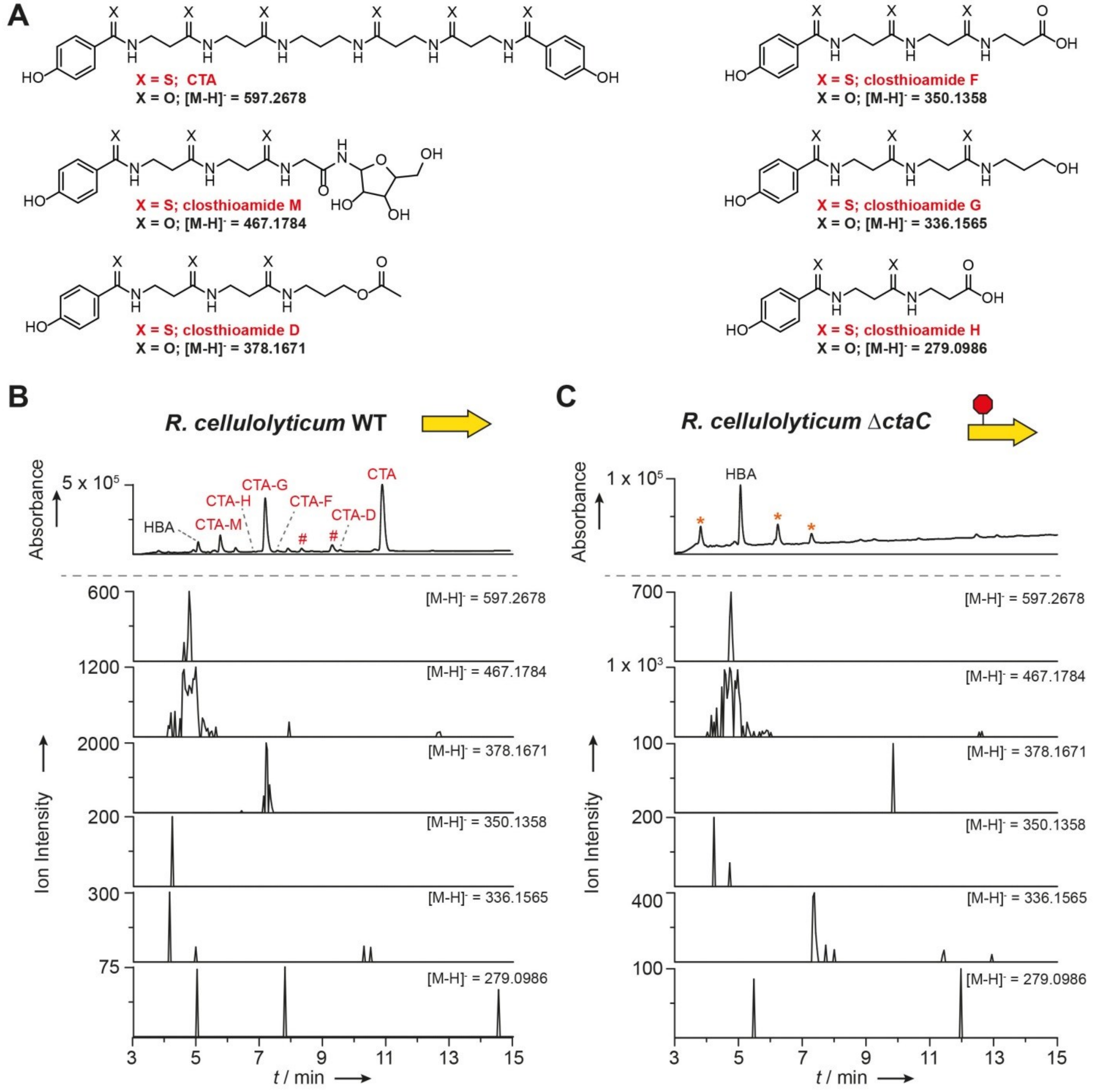

Figure S4. Amidated intermediates are not produced by *Escherichia coli* pCTA-Δ*ctaC*. (A) Structures of CTA intermediates produced by pCTA-expressing *Escherichia coli*[2] along with their corresponding oxygen isosteres. The expected *m/z* value for the $[M-H]^-$ ionic species is displayed. **(B, C)** Extracted ion chromatograms (EICs) corresponding to the compounds displayed in panel A are displayed for organic extracts of cultures of *E. coli* strains expressing either a wild-type CTA gene cluster **(B)** or a gene cluster lacking *ctaC* **(C)**. All EICs are displayed with *m/z* values ± 5 ppm from the calculated exact mass for the compound.

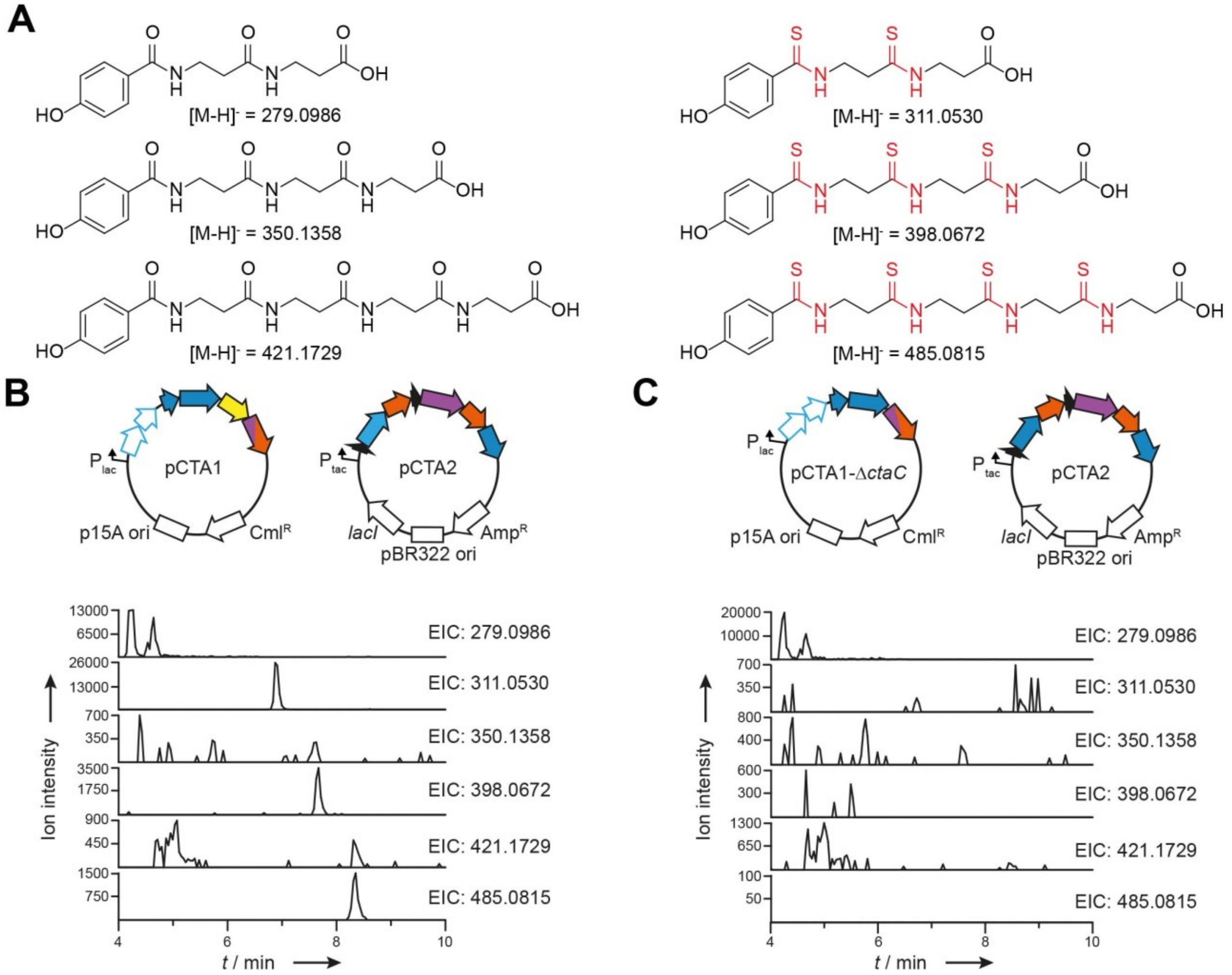

Figure S5. SDS-PAGE analysis of purified proteins. Coomassie stained SDS-protein gels of purified **(A)** MBP-tagged CtaC proteins and **(B)** His_6-tagged PCP proteins are displayed. The expected size of each protein is indicated.

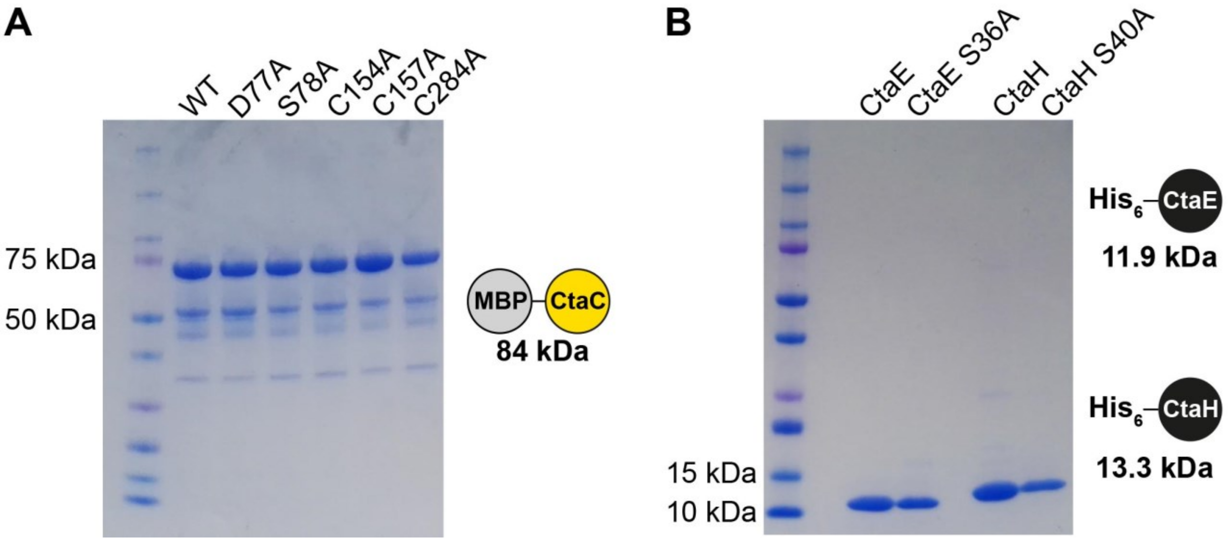

Figure S6. Multiple sequence alignment of CtaC and TtuA/TtcA-type AANH enzymes. Domain structures of TtcA, TtuA and CtaC along with a multiple sequence alignment of members of these AANH subfamilies. The residues responsible for Zn, ATP and Fe-S cluster binding are colored according to the legend and residues selected for mutation in CtaD are colored red.

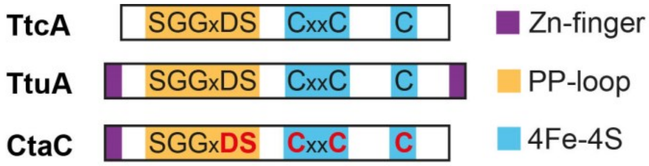


```
CtaC              MIECEVKCKKCLKQIPVDSEGKSMAKNGLCLECYNDELKAQSIDWMEKEQELQEKIEEVR 60
TtcA_EGT70038.1   -------------MSQNQEIS---KKEQYNLNKLQK--------RLRR--NVGEAIADFN 34
TtcA_KEA24785.1   -------------------M---GTLSVNQNKLQK--------RLRR--LAGEAITDFN  27
TtuA_5B4F         -----MVCKVCGQKAQVEMRS---RGLALCREHYLD--------WFVK--ETERAIRRHR 42
TtuA_ADU52157.1   -----MKCTKCGEQAVVPIRR---HNAAFCTEHFFE--------YFEN--QVQRAIKEHR 42
                                                :  .         : .      . *   .

CtaC              RAKGTFDGLVMLSGGKDSIYVAYILSKVYK-----LNIVGLTIDNGFEYQST--FDNSYD 113
TtcA_EGT70038.1   MIEEGDRIMVCLSGGKDSYTMLEIILRNLQQSAPINFSLVAVNLDQKQPGFP---EHVLPE 91
TtcA_KEA24785.1   MIEDGDKVMVCLSGGKDSYTMLDILLYLQKVAPIRFEIVAVNMDQKQPGFP---EHVLPE 84
TtuA_5B4F         MLLPGERVLVAVSGGKDSLALWDVLSRLGYQAVG------LHIELGIGEYSKRSLEVTQA 96
TtuA_ADU52157.1   MFTHDDRILVAVSGGKDSLAIWDVLLRLGYEAHG------LYIDLGIGEYSKRSTEKVRR 96
                          :* :******  :  :*  :            : ::           .

CtaC              IAKKLGISHFIYRLPFEDMREYYNFLLTEDTLKLQDCSQLCFFCGRLLKTISIKVAKMLN 173
TtcA_EGT70038.1   YLEKLGVEYKI-----V---EENTYGIVK--EKIPEGKTTCSLCSRLRRGILYRTATELG 141
TtcA_KEA24785.1   YLKSIGVEYHI-----V---EKDTYSVVK--EKIPEGKTTCSLCSRLRRGTLYTFADEIG 134
TtuA_5B4F         FARERGLELLV-----VDLKEAYGFGVPE--LARLSGRVACSACGLSKRYIINQVAVEEG 149
TtuA_ADU52157.1   FAEERGAVLRV-----VSVEEEYGLGTTE--LARKAGRPACSACGLNKRYLFNREALAGG 149
                    .. *    :         *       :          *  *.   :      *   .

CtaC              VTAVFSGHTLEQIRALGDEEGKDKSFDIRRRYIQNYSVQNYKKALDLLKEKSKESISYLF 233
TtcA_EGT70038.1   ATKIALGHHRDDILQTLFLNM----FYG-----------GKMKGMPPKLM---------- 176
TtcA_KEA24785.1   ATKMALGHHRDDILETFFLNM----FYG-----------GTLKAMPPKLL---------- 169
TtuA_5B4F         FRVVATGHNLDDEAAVLFGNL----LNPQE---------ETLSRQGPVLP---------- 186
TtuA_ADU52157.1   YDVIVTGHNLDDEAATLLGNV----LHWNT---------GYLARQAPVLEA--------- 187
                     :  **  ::       :     :

CtaC              DDNLETLNFDRFIYPLQYFEYKPIEIVELLSKELGWVADTHFTKKYIS-SGCKLGKLMEY 292
TtcA_EGT70038.1   ----SDDGKHIVIRPLAYCREKDIQRFADAKA-FP----------IIPCNLCGSQPNLQ- 220
TtcA_KEA24785.1   ----ADDGRNVVIRPLAYCSEKDIEAYSQLKE-FP----------IIPCNLCGSQENLQ- 213
TtuA_5B4F         ----EKPGLAARVKPFYRFSEREVLSYTLLRG-IR----------YLH-EECPNAKGAK- 229
TtuA_ADU52157.1   ----SAPNLVKKVKPLYRLTEREIASYAVLRG-ID----------YIV-EECPMAKGAK- 230
                        .    : *:     : :         :             :  . *       :

CtaC              IATKNGSTTYVEREFSDQIRRGSLS  317
TtcA_EGT70038.1   --------RQVIADMLRDWDKRYPG  237
TtcA_KEA24785.1   --------RQVVKEMLLEWERKSPG  230
TtuA_5B4F         --------SLLYKEALNLVERSMPG  246
TtuA_ADU52157.1   --------SIQYKHILNQLEELSPG  247
                          .     .   .
```

Figure S7. Phosphopantetheinylation of *apo*-CtaE and *apo*-CtaH *in vitro* by Sfp. MALDI-TOF-MS spectral overlays of **(A)** CtaE and **(B)** CtaH phosphopantetheinylation assays with multiple coenzyme A (CoA) derivatives. The expected mass shift relative to *apo*-PCP for each CoA derivative is listed in parentheses. **(C)** A multiple sequence alignment of CtaE/H and diverse acyl and peptidyl carrier proteins is displayed. The conserved phosphopantetheinyl transferase recognition site is indicated by the box. The serine residue expected to be the site of phosphopantethein (PP) attachment on CtaE/H is colored red. In order to localize the site of PP attachment on the PCPs, the conserved serine residue was mutated to alanine for each PCP and the corresponding mutants ($CtaE_{S36A}$ and $CtaH_{S40A}$) were isolated. **(D)** MALDI-TOF-MS spectral overlays of phosphopantetheinylation assays performed with mutant versions of CtaE ($CtaE_{S36A}$) and CtaH ($CtaH_{S40A}$) that lack the conserved serine residue. Consistent with the prediction, these mutant proteins were not processed by Sfp.

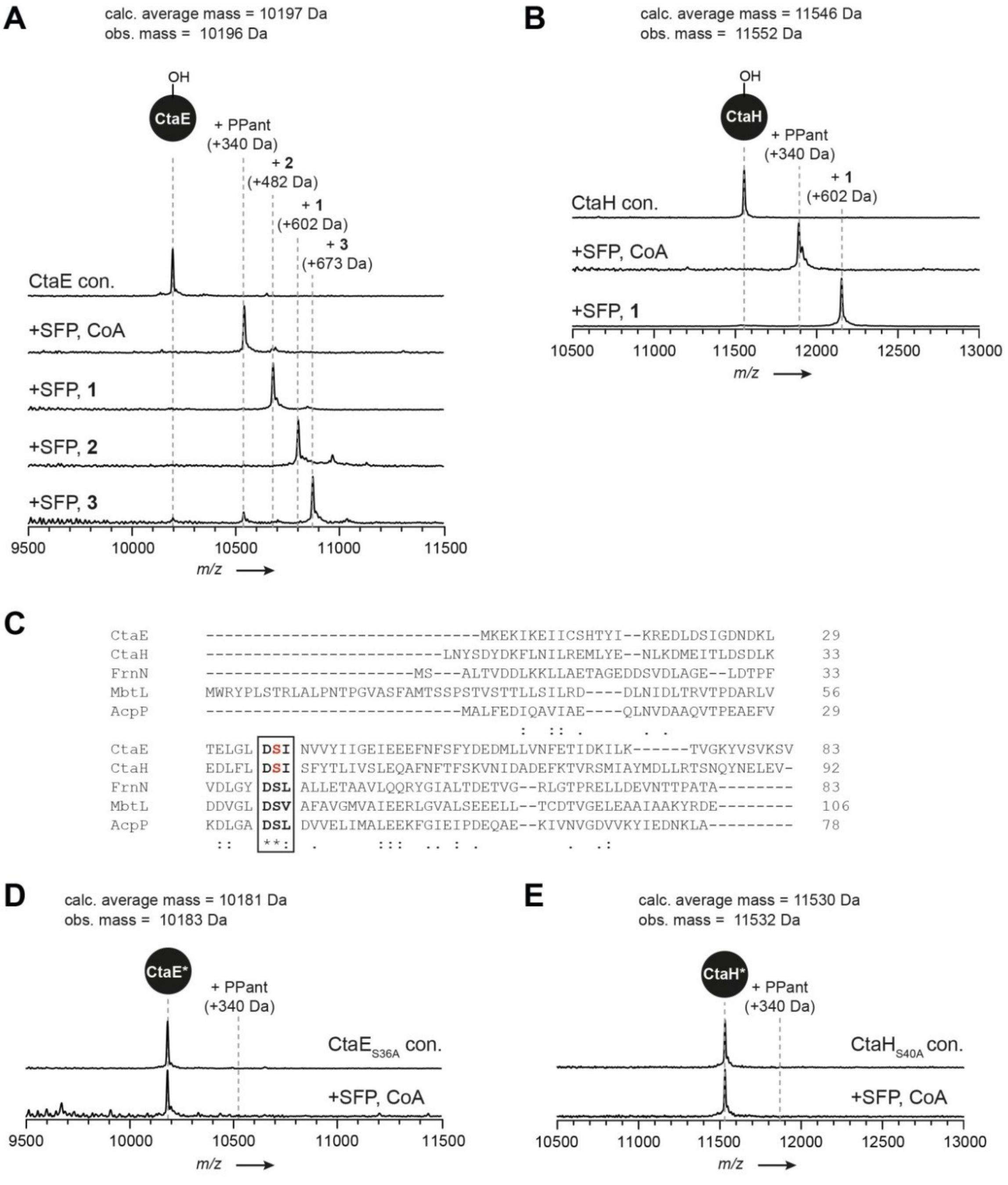

Figure S8. Substrates presented on CtaH are not recognized by CtaC. Representative MALDI-TOF-MS spectral overlay of CtaC reactions with **1**-*holo*-CtaH. Spectra were calibrated to the mass of the unmodified PCP. Guide lines denote the expected mass shifts (+ 16 Da) for substitution of oxygen by sulfur.

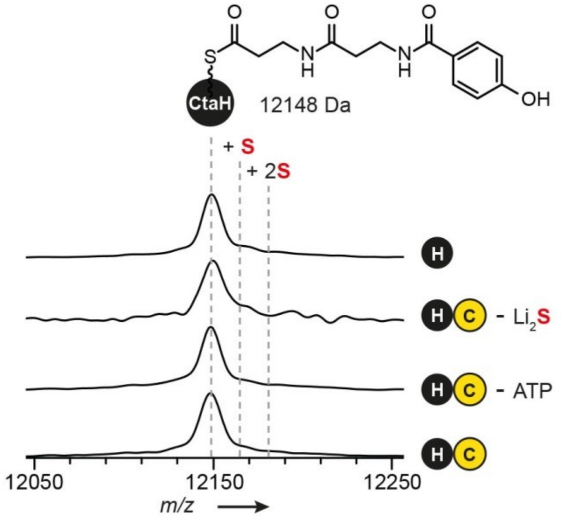

Figure S9. CtaC does not recognize substrates lacking the HBA endcap. Representative MALDI-TOF-MS spectral overlay of CtaC reactions with **2**-*holo*-CtaE. Spectra were calibrated to the mass of the unmodified PCP. Guide lines denote the expected mass shifts (+ 16 Da) for substitution of oxygen by sulfur.

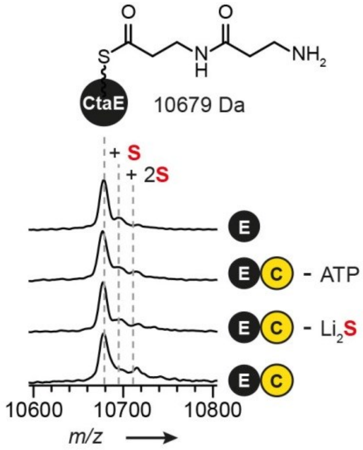

Figure S10. LC-HR-MS/MS analysis of thioamidated product from 1-*holo*-CtaE reaction. (A) HR-MS spectrum of the **1**-*holo*-CtaE/CtaC reaction product. The skewed isotope pattern is consistent with the presence of multiple sulfur atoms. **(B)** The structure of di-thioamidated **1** along with the HRMS-MS spectrum and expected masses of assignable fragments. Ions that were detected in the HRMS-MS spectrum are colored blue. Asterisks denote ions formed by neutral loss of H_2, while masses in parentheses correspond to ions formed by neutral loss of H_2S.

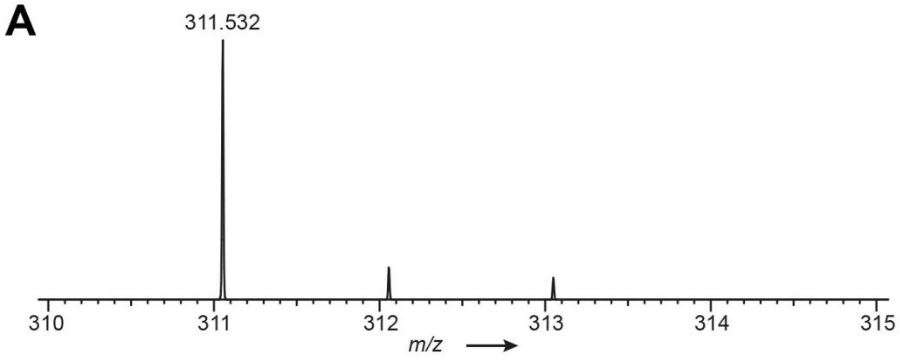

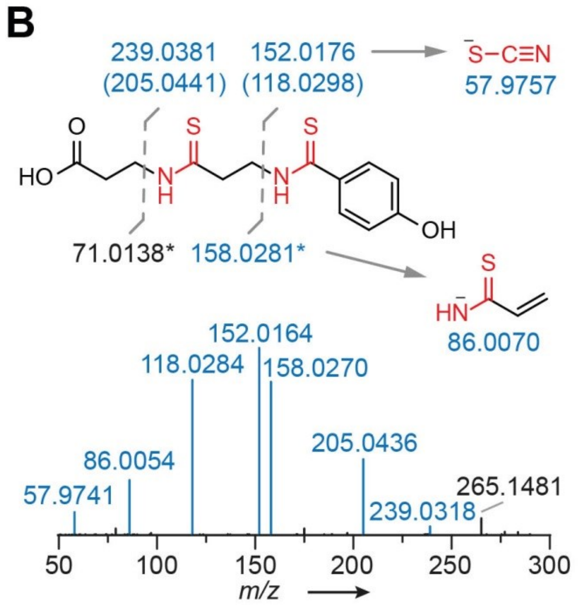

Figure S11. CtaC activity test with 1. LC-HR-MS profile of thioamidation reactions performed with **1** along with the structure of the compound and the exact mass of the [M-H]$^-$ ionic species of **1** and its thioamidated derivatives. All extracted ion chromatograms (EICs) are displayed with *m/z* values ± 5 ppm from the calculated exact mass for the compound. Abs., absorbance.

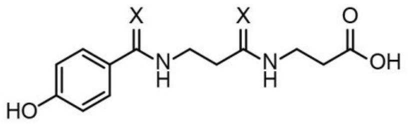

X	[M-H]$^-$
2 x O , 0 x S	279.0986
1 x O , 1 x S	295.0758
0 x O , 2 x S	311.0530

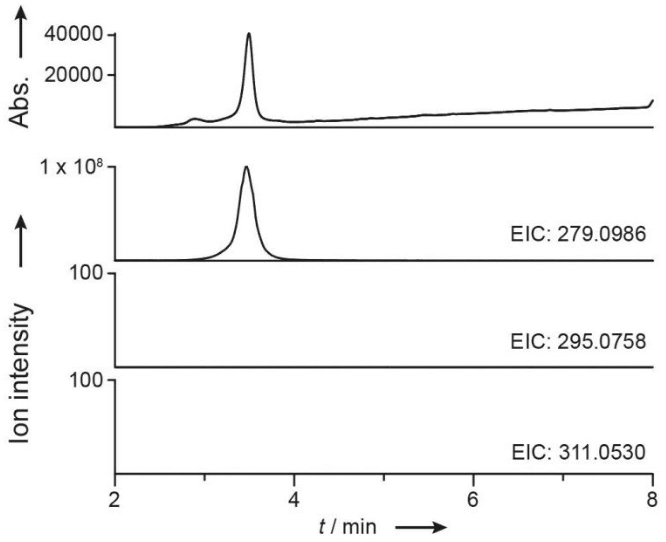

Figure S12. CtaC activity test with 1-CoA. LC-HR-MS profile of thioamidation reactions performed with **1**-CoA along with the structure of the compound and the exact mass of the [M-H]$^-$ ionic species of **1**-CoA and its thioamidated derivative. All extracted ion chromatograms (EICs) are displayed with *m/z* values ± 5 ppm from the calculated exact mass for the compound. Abs., absorbance.

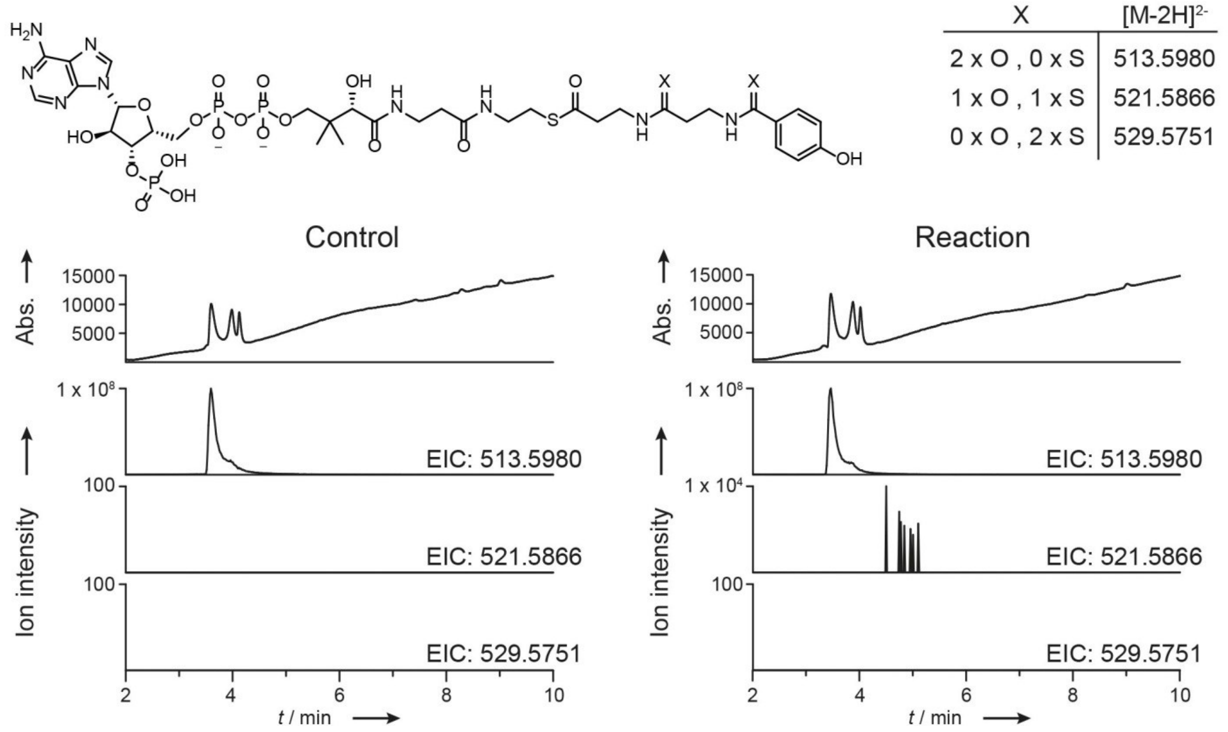

Figure S13. CtaC activity test with 3-CoA. LC-HR-MS profile of thioamidation reactions performed with **3**-CoA along with the structure of the compound and the exact mass of the $[M-H]^-$ ionic species of **3**-CoA and its thioamidated derivatives. All extracted ion chromatograms (EICs) are displayed with *m/z* values ± 5 ppm from the calculated exact mass for the compound. Abs., absorbance.

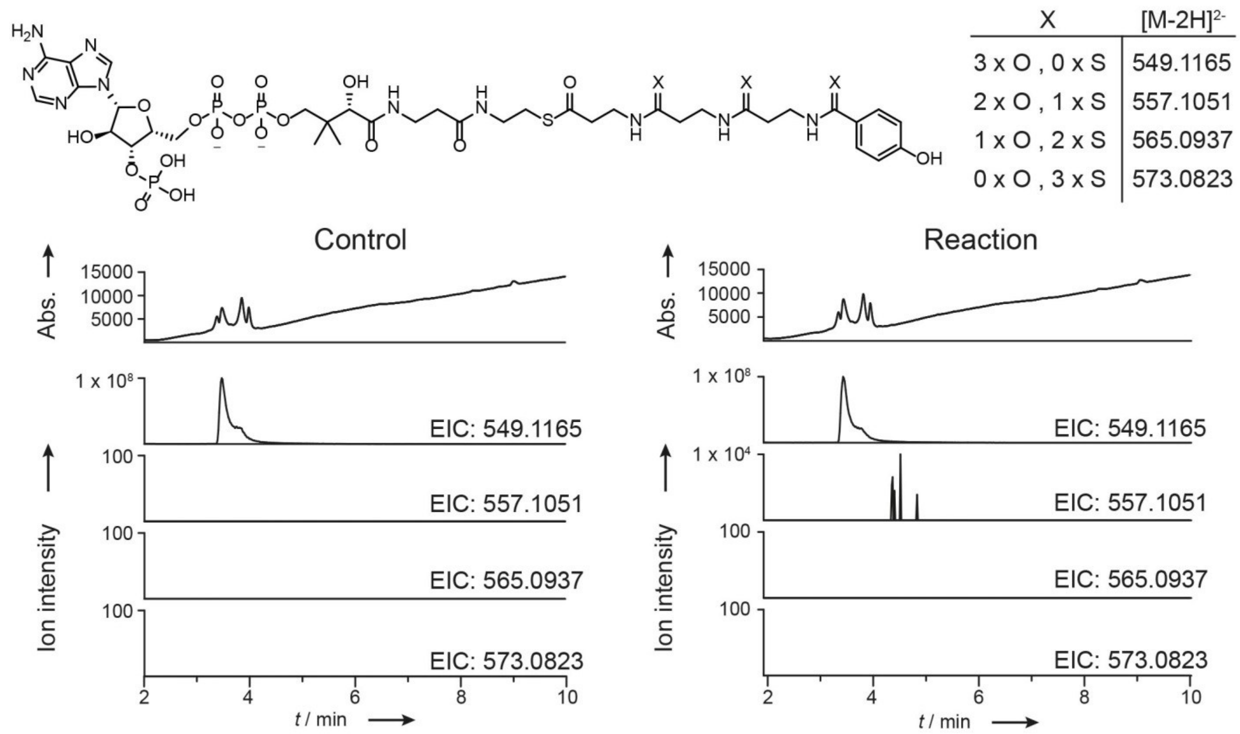

Figure S14. CtaC activity test with 4. LC-HR-MS profile of thioamidation reactions performed with **4** along with the structure of the compound and the exact mass of the [M-H]$^-$ ionic species of **4** and its thioamidated derivative. All extracted ion chromatograms (EICs) are displayed with *m/z* values ± 5 ppm from the calculated exact mass for the compound. Abs., absorbance.

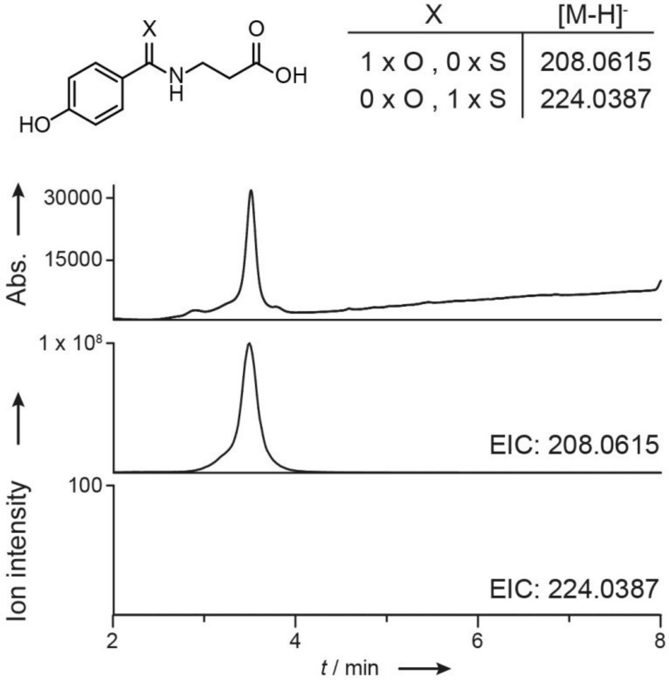

Figure S15. CtaC activity test with 5. LC-HR-MS profile of thioamidation reactions performed with **5** along with the structure of the compound and the exact mass of the [M-H]$^-$ ionic species of **5** and its thioamidated derivatives. All extracted ion chromatograms (EICs) are displayed with *m/z* values ± 5 ppm from the calculated exact mass for the compound. Abs., absorbance.

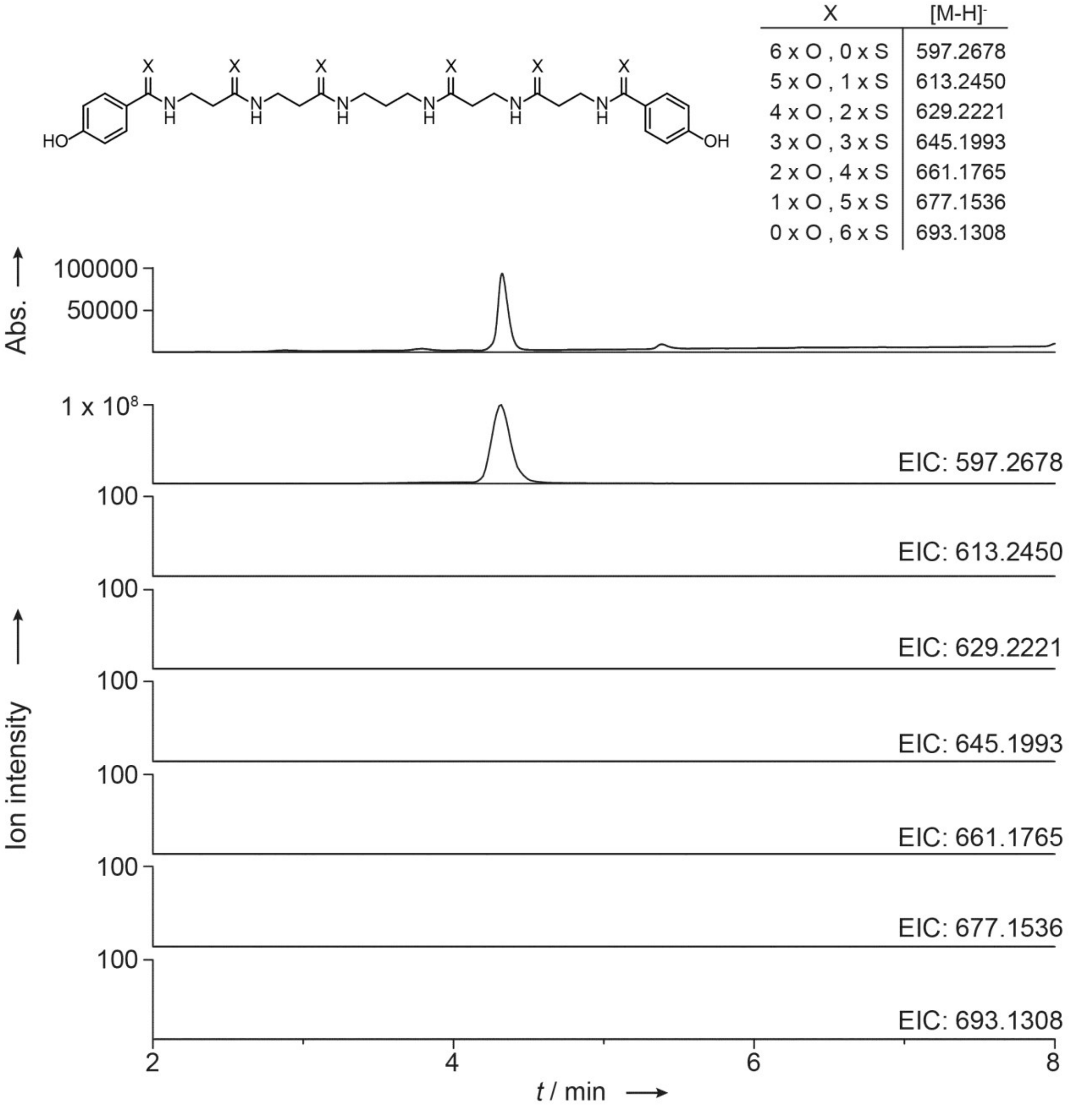

Figure S16. Detection of ATP hydrolysis products from CtaC reactions. HPLC profiles (absorbance at 260 nm) of CtaC reactions with **1**-*holo*-CtaE. The red strikethrough on CtaC denotes reactions performed with heat-inactivated CtaC.

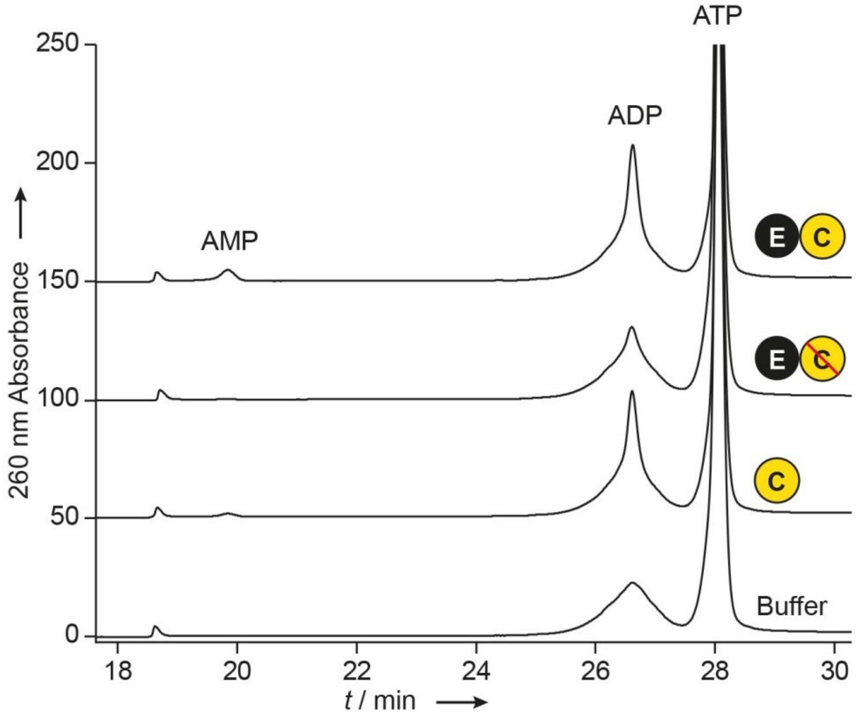

Figure S17. MALDI-TOF spectra of mutant CtaC reactions with 1-*holo*-CtaE. Representative MALDI-TOF-MS spectral overlay of mutant CtaC reactions with **1**-*holo*-CtaE performed for 6 h. Spectra were calibrated to the mass of the unmodified PCP. Guide lines denote the expected mass shifts (+ 16 Da) for substitution of oxygen by sulfur. The red strikethrough on CtaC denotes reactions performed with heat-inactivated CtaC. Significant activity was only observed for the C154A mutant. See **Figure 2E** for MALDI-TOF spectra for reactions performed for 22 h.

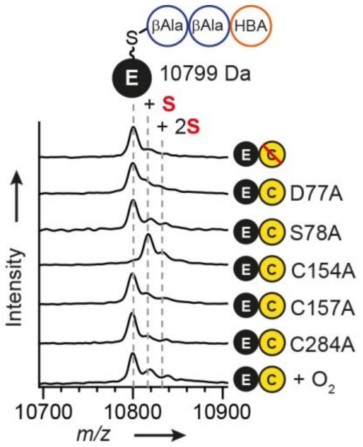

Figure S18. Fe-S cluster binding by CtaC mutants. (A) UV-visible absorbance spectra of the reconstituted mutant CtaC enzymes. For reference, the spectrum of the wild-type enzyme is also shown. **(B)** Iron and sulfur content of the reconstituted CtaC mutants. The error represents the standard deviation from the mean (n = 3).

A

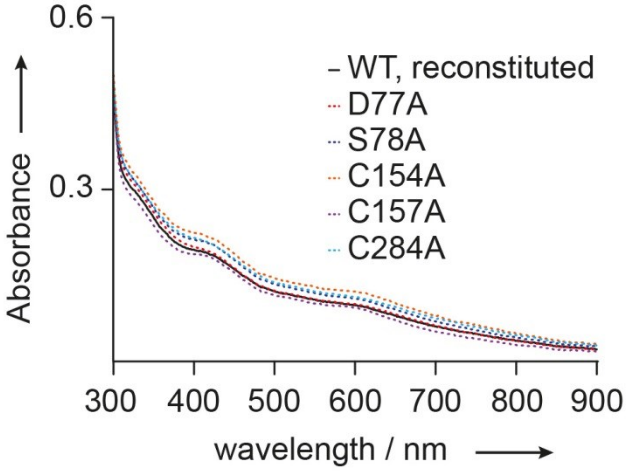

B

	Fe per protomer	S per protomer
CtaC D77A	5.0 ± 0.2	5.0 ± 0.2
CtaC S78A	4.2 ± 0.2	4.8 ± 0.1
CtaC C154A	4.6 ± 0.2	4.9 ± 0.2
CtaC C157A	4.4 ± 0.2	4.5 ± 0.3
CtaC C284A	4.7 ± 0.1	4.8 ± 0.2

Figure S19. Increased processing time does not lead to further processing of 1-*holo*-CtaE by CtaC. Representative MALDI-TOF-MS spectral overlay of CtaC reactions with **1**-*holo*-CtaE performed for 22 h. Spectra were calibrated to the mass of the unmodified PCP. Guide lines denote the expected mass shifts (+ 16 Da) for substitution of oxygen by sulfur.

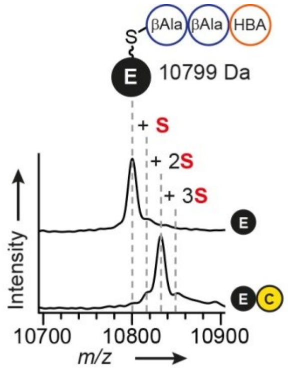

Figure S20. LC-HR-MS analysis of thioamidated product from 3-*holo*-CtaE reaction. (A) LC-HR-MS profiles of thioamidation reactions with **3**-*holo*-CtaE following thioester cleavage. Traces correspond to the extracted ion chromatogram of the $[M\text{-}H]^-$ ionic species for tri-thioamidated **3** and are displayed with *m/z* values ± 5 ppm from the calculated exact mass. **(B)** HR-MS spectrum of the **3**-*holo*-CtaE/CtaC reaction product. The skewed isotope pattern is consistent with the presence of multiple sulfur atoms. **(C)** A HRMS-MS spectrum of the product of the **3**-*holo*-CtaE/CtaC reaction. The structure of tri-thioamidated **3** is displayed with the expected masses of the assignable fragments. Ions that were detected in the HRMS-MS spectrum are colored blue. Asterisks denote ions formed by neutral loss of H_2, while masses in parentheses correspond to ions formed by neutral loss of H_2S.

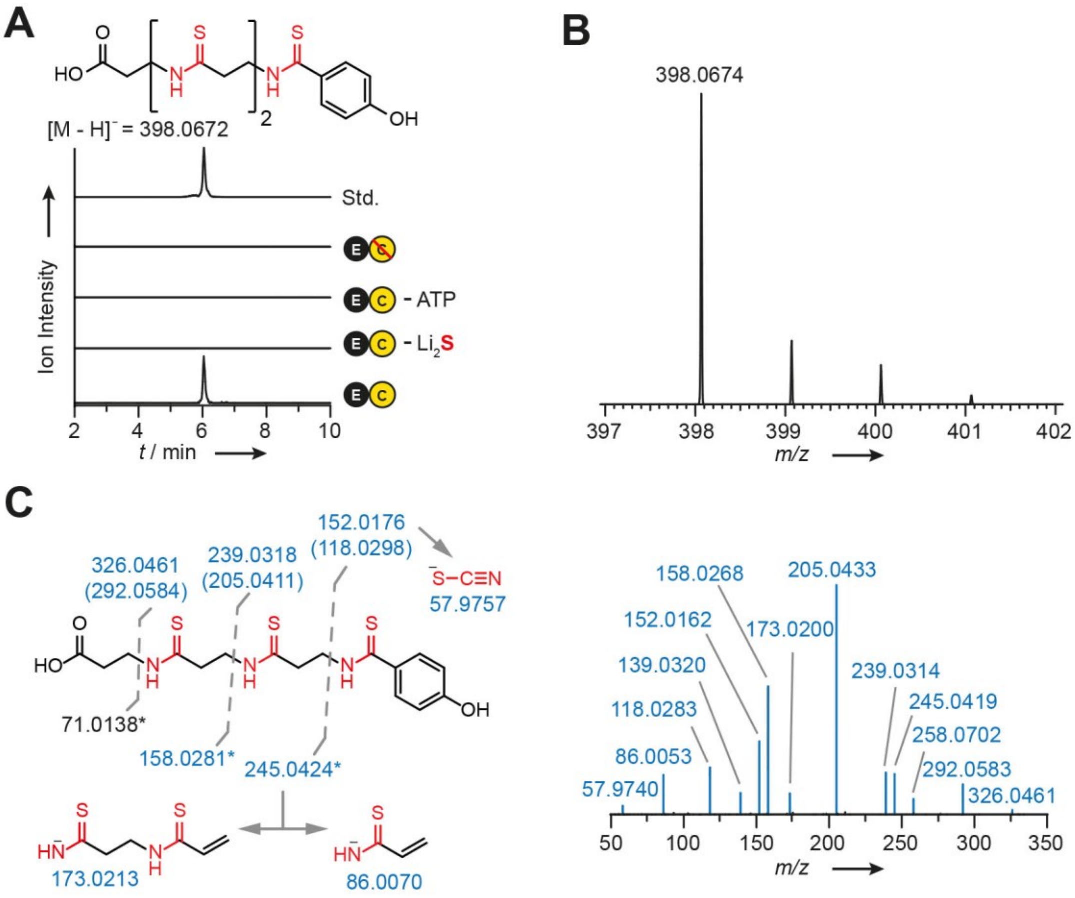

Figure S21. ^{1}H-NMR spectrum of compound 1.

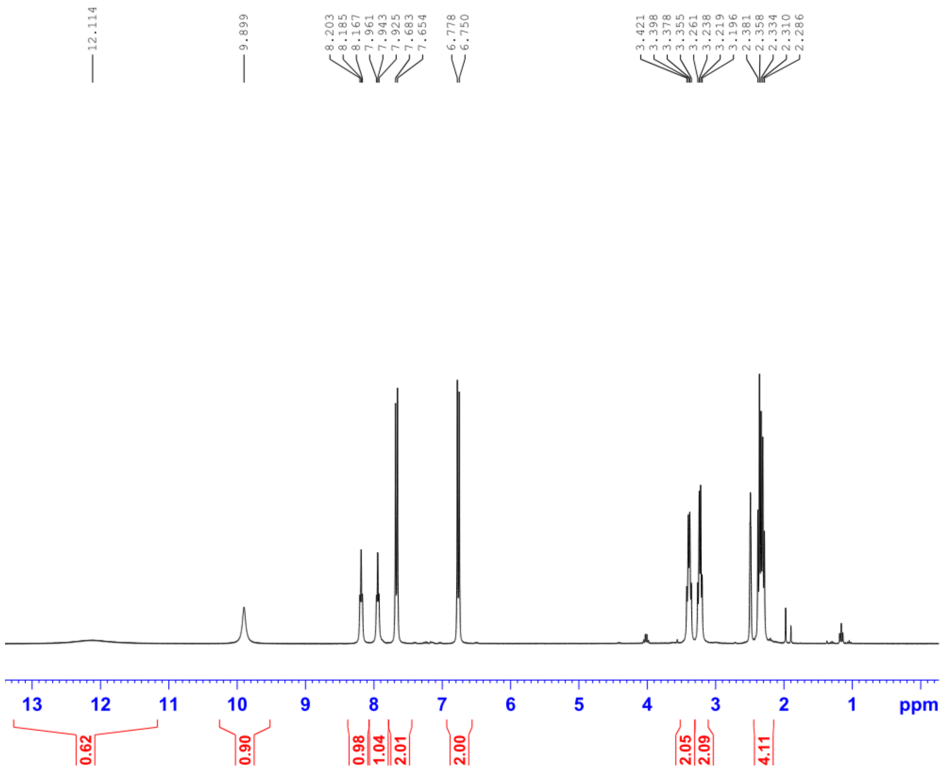

Figure S22. ^{13}C-NMR spectrum of compound 1.

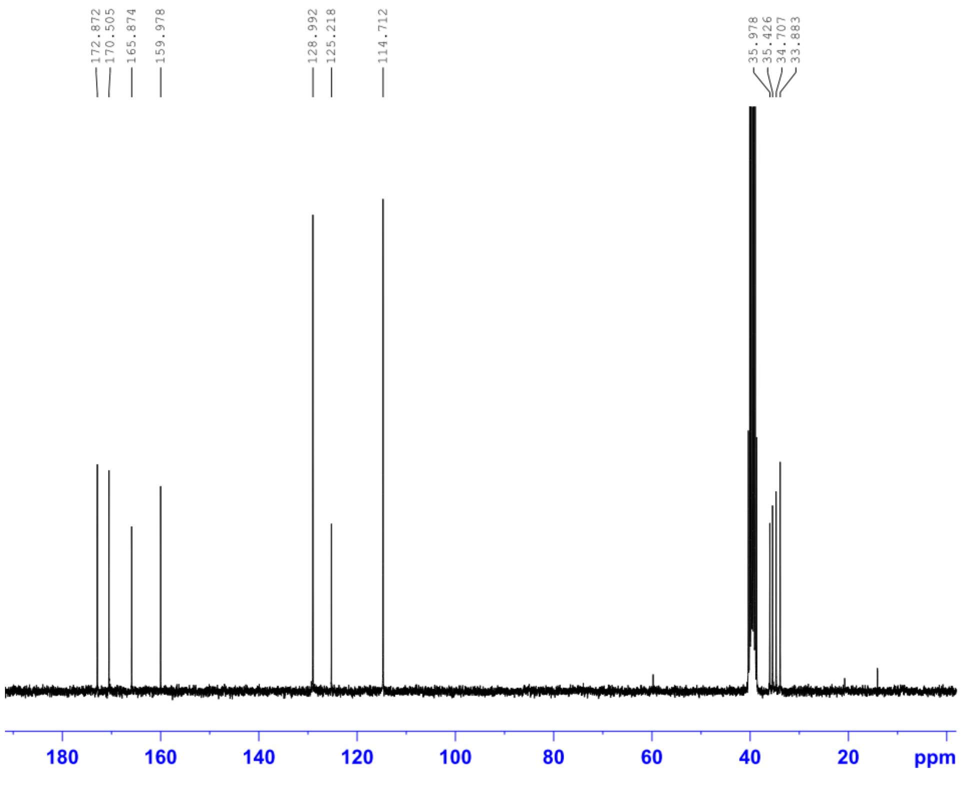

Figure S23. ^{1}H-NMR spectrum of 1-CoA.

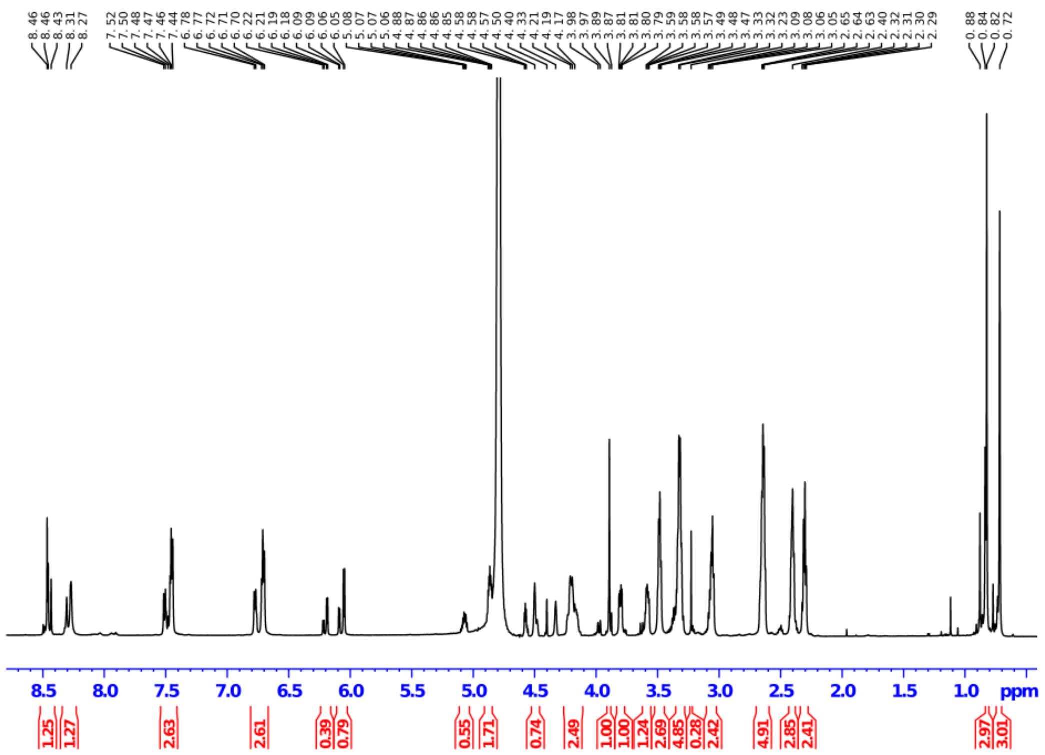

Figure S24. ^{13}C-NMR spectrum of 1-CoA.

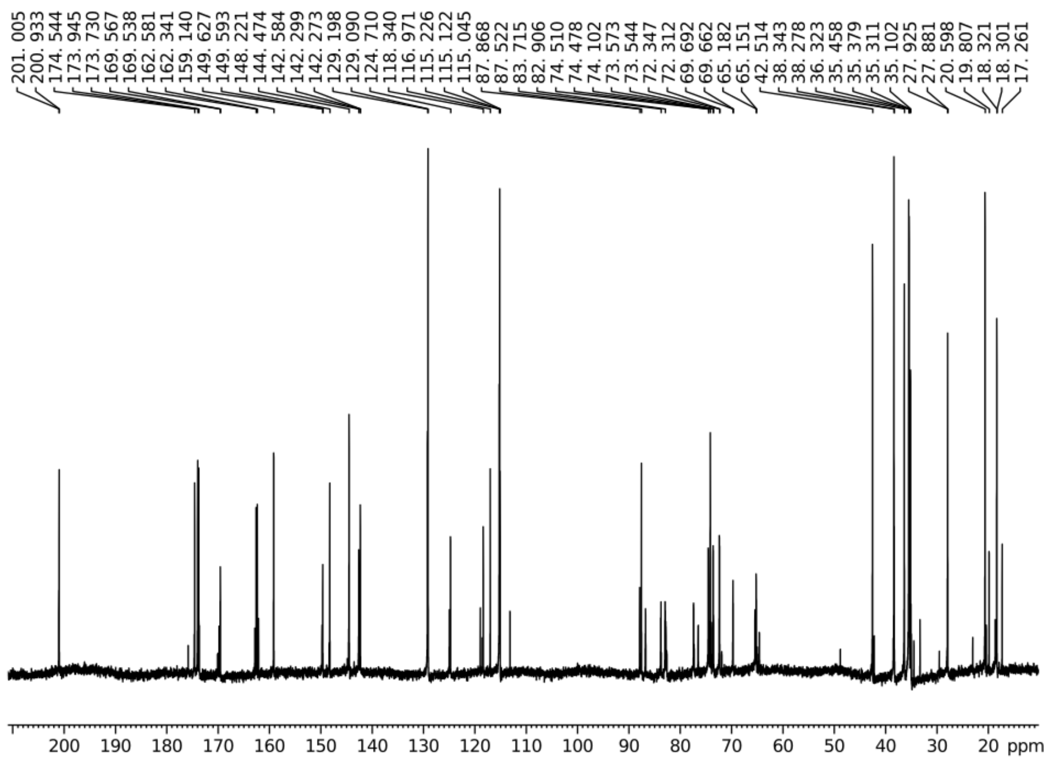

Figure S25. **^{1}H-NMR spectrum of 2-CoA.**

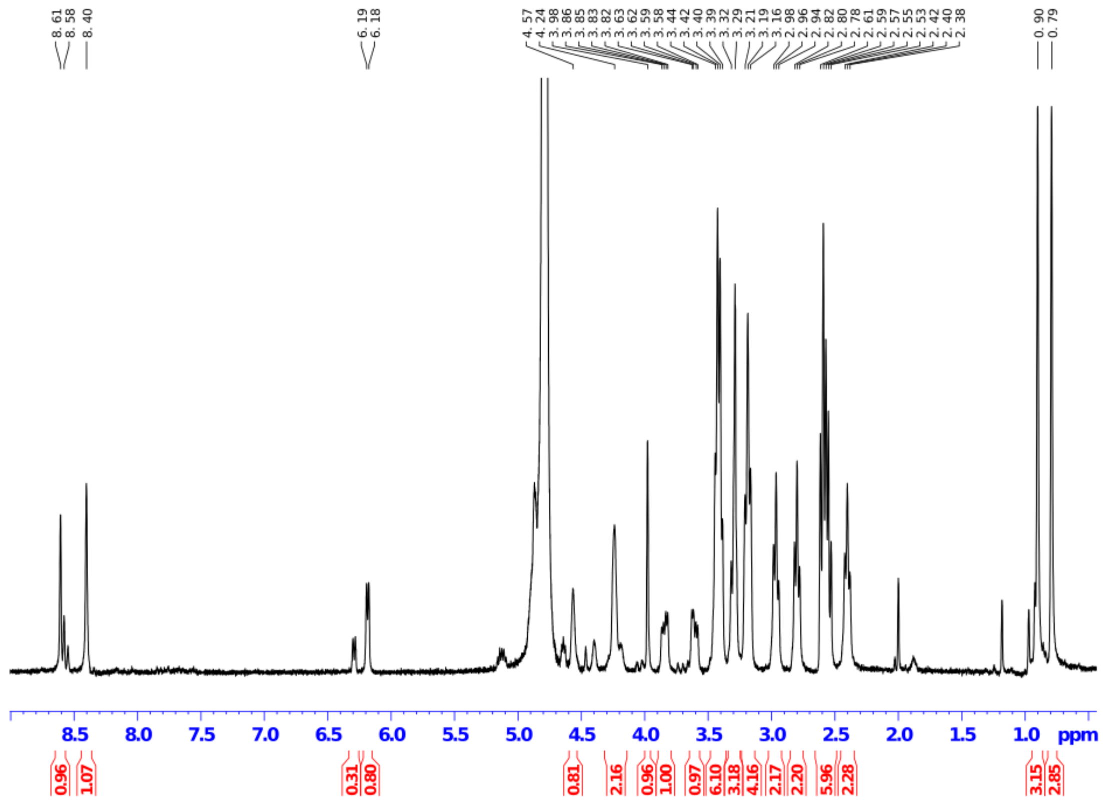

Figure S26. **^{13}C-NMR spectrum of 2-CoA.**

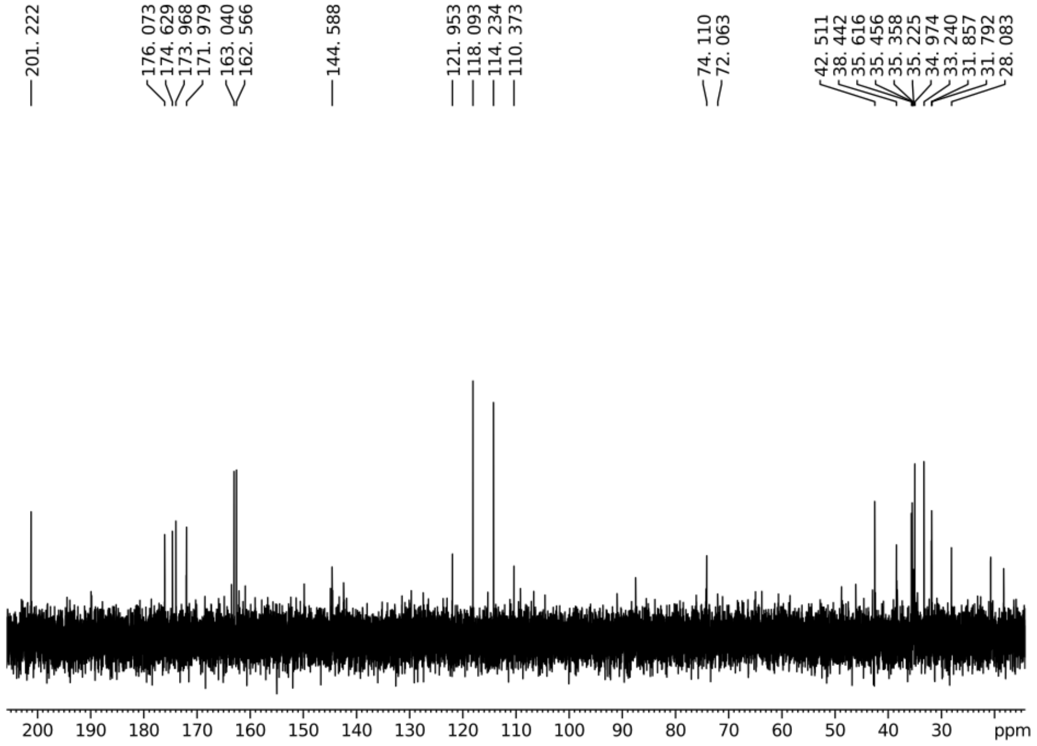

Figure S27. ^{1}H-NMR spectrum of 3-CoA.

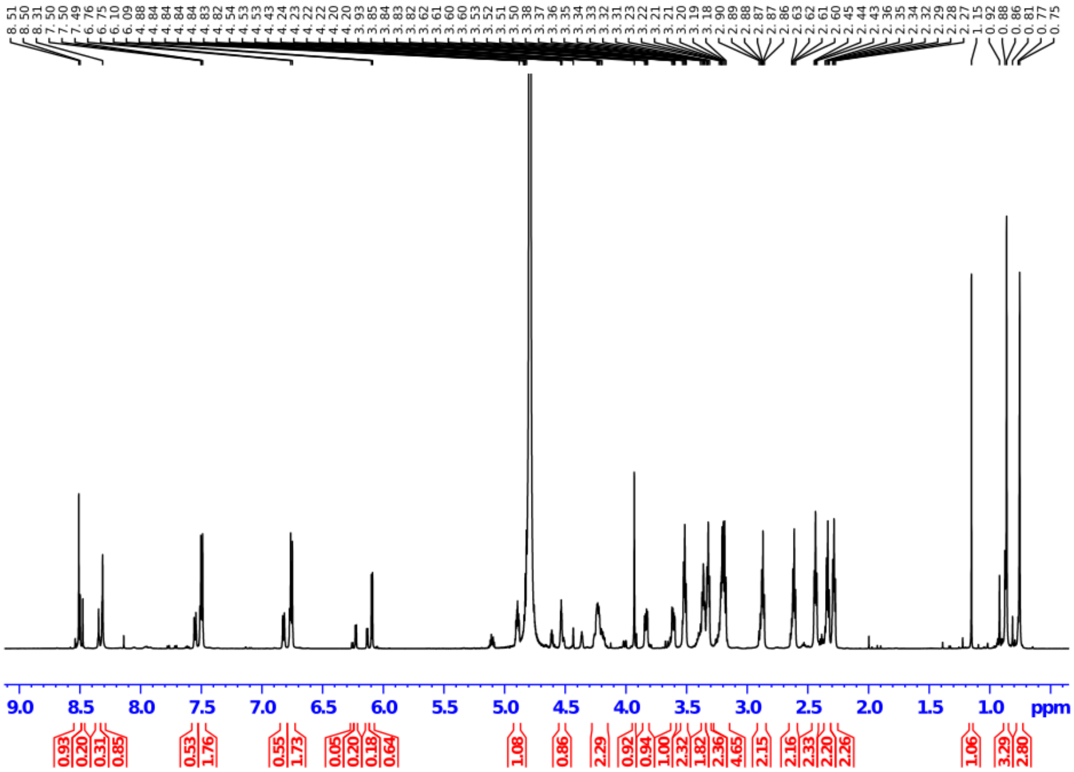

Figure S28: ^{13}C-NMR of 3-CoA.

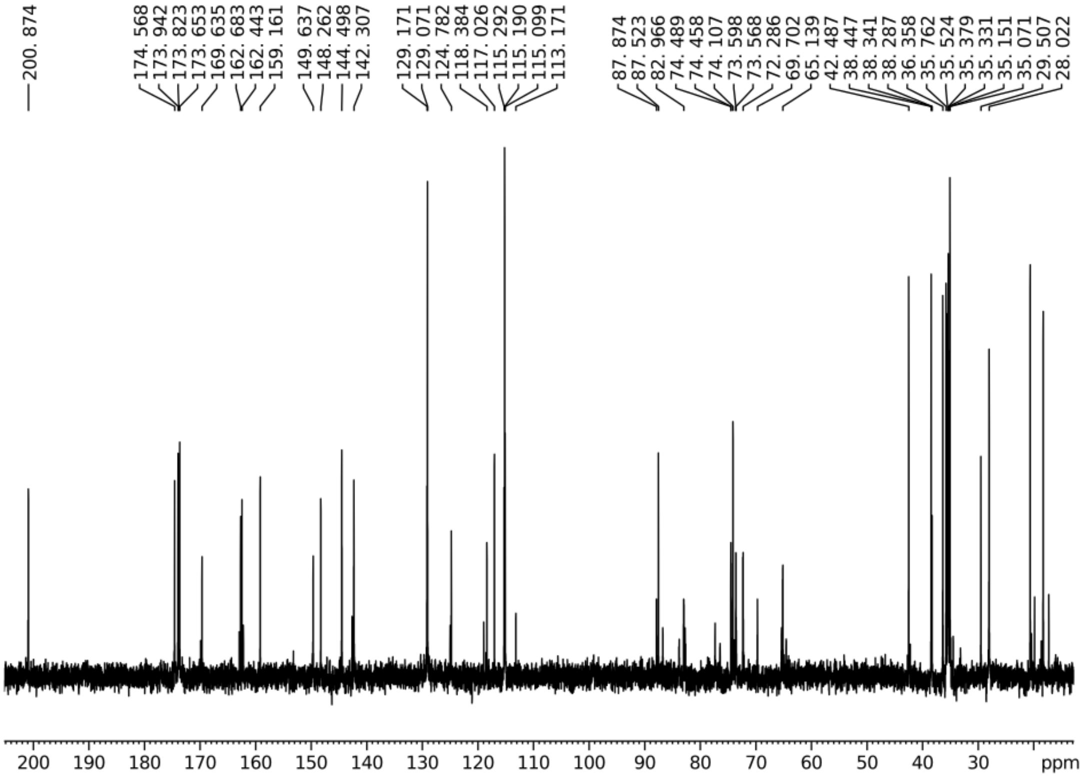

Figure S29. ^{1}H-NMR spectrum of compound 4.

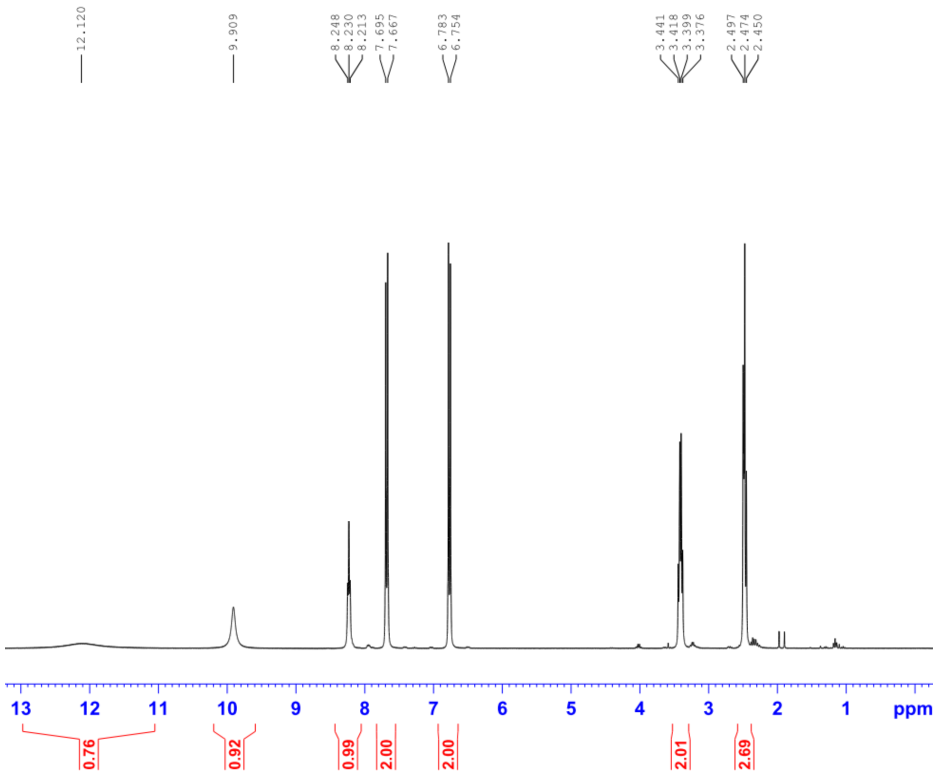

Figure S30. ^{13}C-NMR spectrum of compound 4.

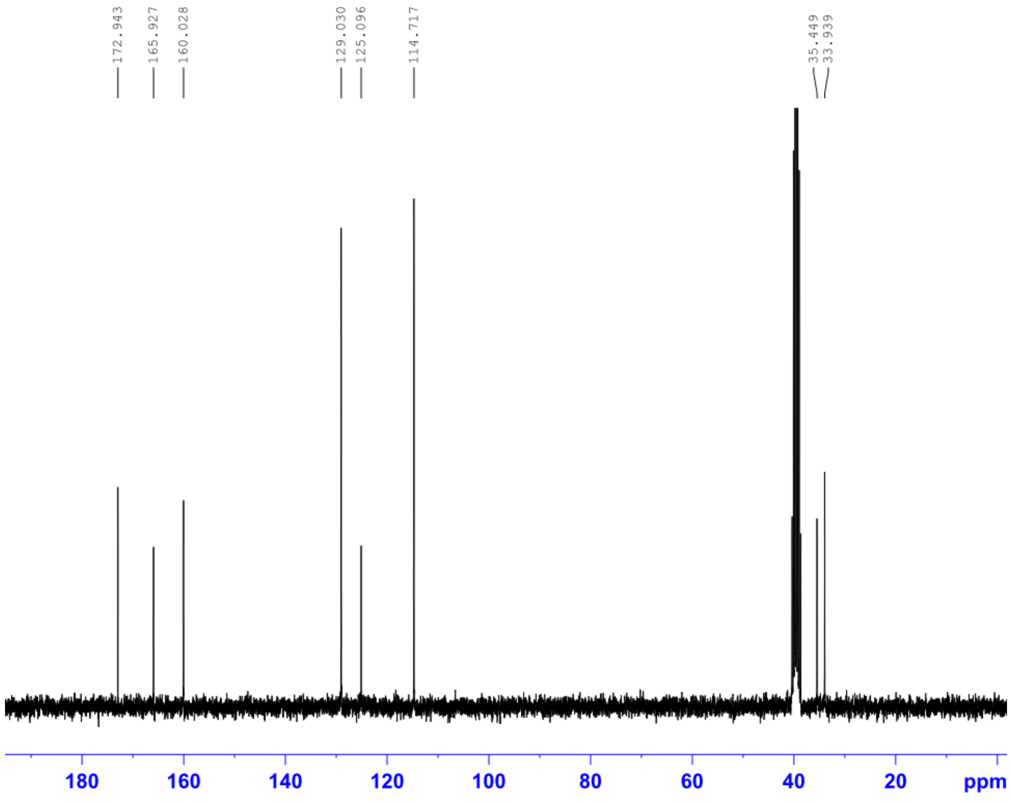

Figure S31. ¹H-NMR spectrum of compound 9.

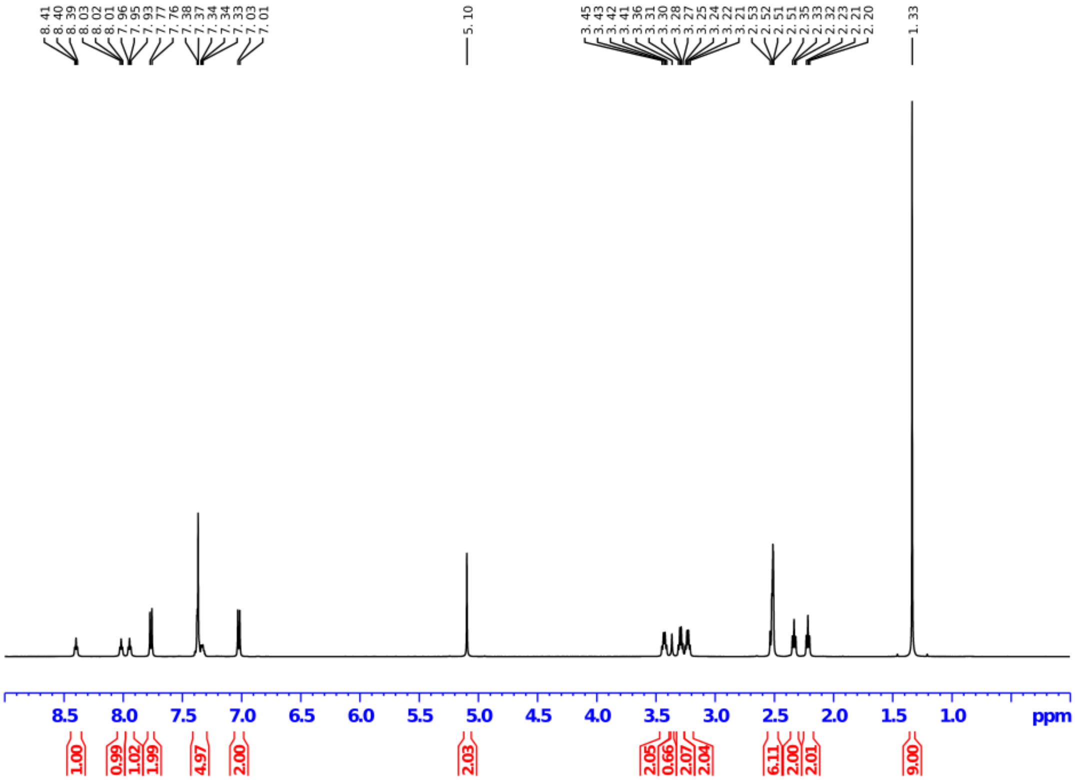

Figure S32. ¹³C-NMR spectrum of compound 9.

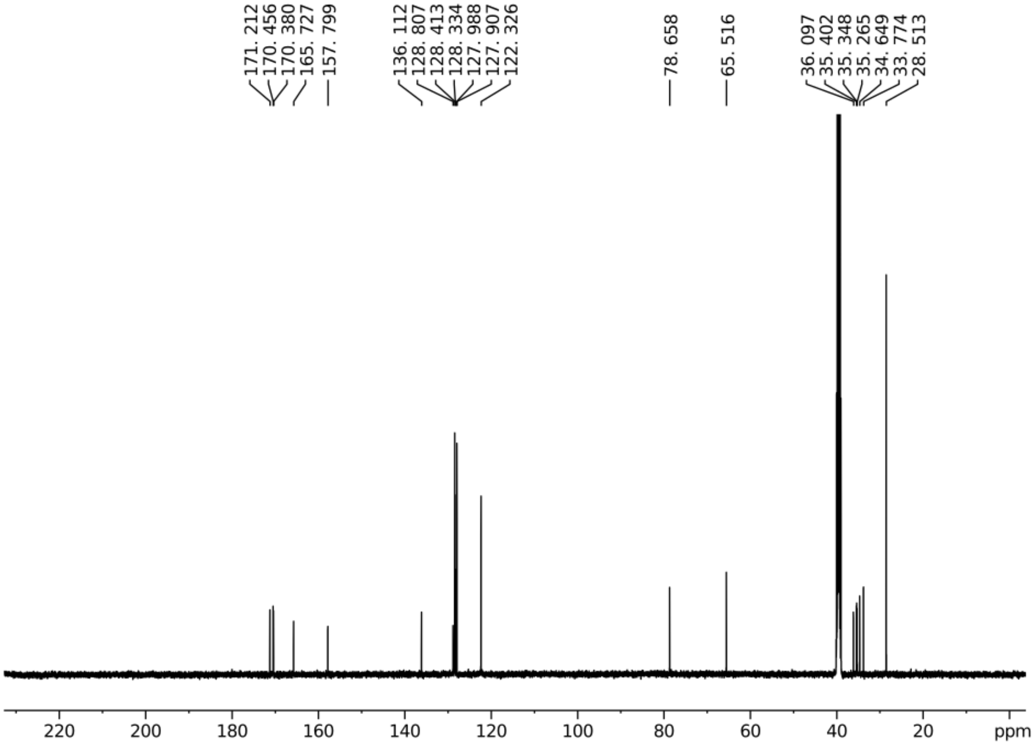

Figure S33. ^{1}H-NMR spectrum of compound 10.

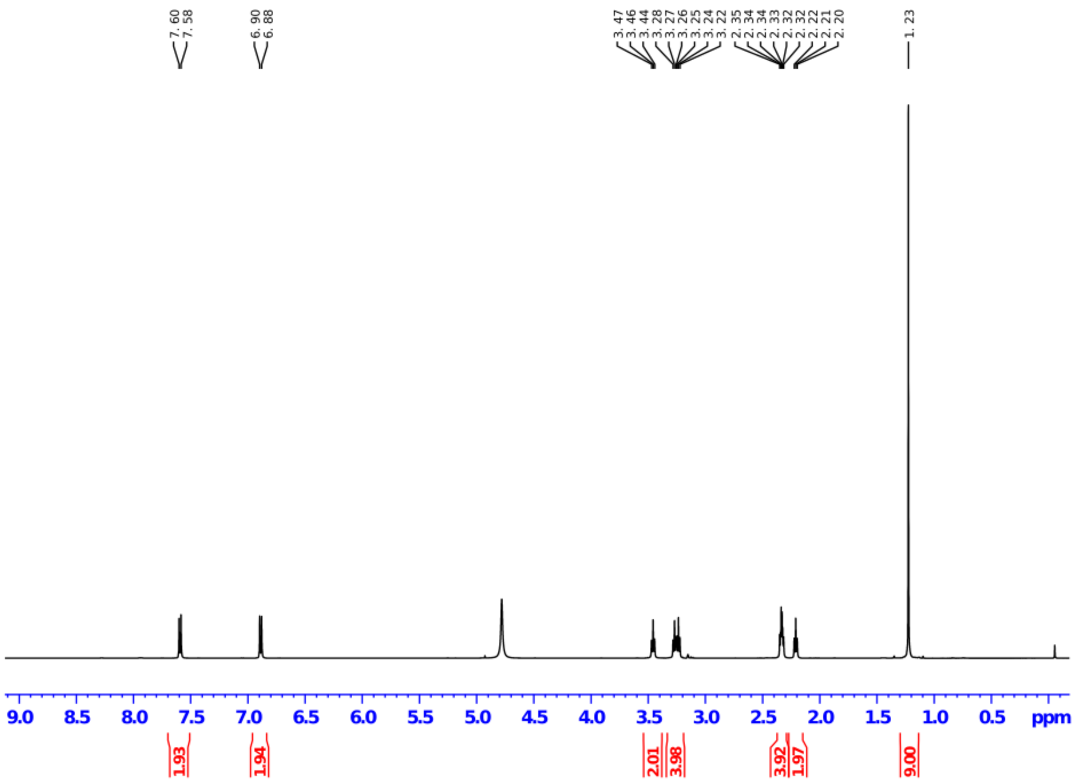

Figure S34. ^{13}C-NMR spectrum of compound 10.

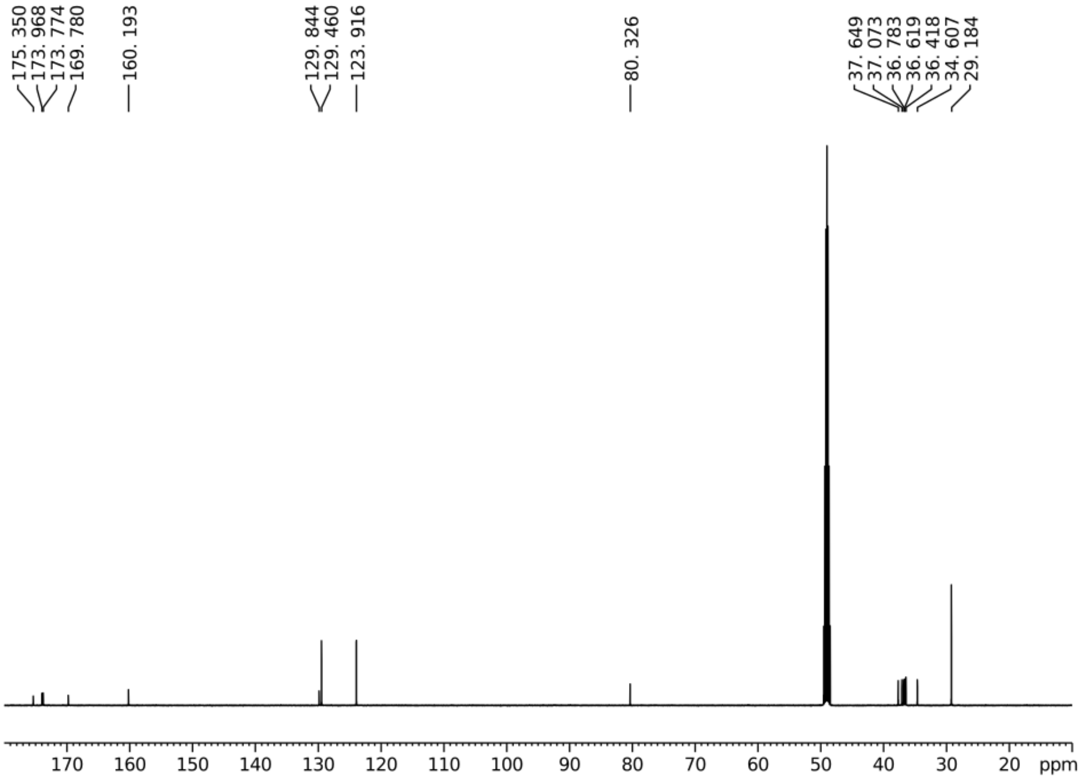

Table S1. Strains used in this study.

Strains	Relevant genotype and descriptions	Source
Ruminiclostridium cellulolyticum	Wild type (DSM 5812)	DSMZ
Ruminiclostridium cellulolyticum Δ*ctaC*	CRISPR/Cas inactivated Ccel_3258	This study
Escherichia coli TOP10	General cloning strain	Laboratory strain
Escherichia coli Rosetta (DE3)	Protein expression strain	NEB
E. coli Rosetta (DE3) pET28-*ctaC*	Strain contains pET28-*ctaC*	This study
E. coli Rosetta (DE3) pMAL-*ctaC*	Strain contains pMAL-*ctaC*	This study
E. coli Rosetta (DE3) pMAL-$ctaC_{\mathrm{D77A}}$	Strain contains pMAL-$ctaC_{\mathrm{D77A}}$	This study
E. coli Rosetta (DE3) pMAL-$ctaC_{\mathrm{S78A}}$	Strain contains pMAL-$ctaC_{\mathrm{S78A}}$	This study
E. coli Rosetta (DE3) pMAL-$ctaC_{\mathrm{C154A}}$	Strain contains pMAL-$ctaC_{\mathrm{C154A}}$	This study
E. coli Rosetta (DE3) pMAL-$ctaC_{\mathrm{C157A}}$	Strain contains pMAL-$ctaC_{\mathrm{C157A}}$	This study
E. coli Rosetta (DE3) pMAL-$ctaC_{\mathrm{C284A}}$	Strain contains pMAL-$ctaC_{\mathrm{C284A}}$	This study
E. coli Rosetta (DE3) pET28-*ctaE*	Strain contains pET28-*ctaE*	This study
E. coli Rosetta (DE3) pET28-$ctaE_{\mathrm{S36A}}$	Strain contains pET28-$ctaE_{\mathrm{S36A}}$	This study
E. coli Rosetta (DE3) pET28-*ctaH*	Strain contains pET28-*ctaH*	This study
E. coli Rosetta (DE3) pET28-$ctaH_{\mathrm{S40A}}$	Strain contains pET28-$ctaH_{\mathrm{S40A}}$	This study
Escherichia coli HM0079	Strain contains a chromosomal copy of *sfp* from *Bacillus subtilis*	[15]
E. coli HM0079 pCTA1 pCTA2	Strain contains pSU18-CTA1 and pTrc99a-CTA2 for expression of whole CTA gene cluster	[2]
E. coli HM0079 pCTA1-Δ*ctaC* pCTA2	Strain contains mutant version of pSU18-CTA1 lacking *ctaC*	This study

Table S2. Plasmids used in this study.

Plasmid	Description	Source
pCasC	pSOS-Cas-Gm with D10A Cas9 mutation; Clostridia-adapted vector for CRISPR/Cas editing in *R. cellulolyticum*	[2]
pUC-*ctaC*-target	Contains sgRNA cassette (P4 promoter, sgRNA, spy terminator), N20 sequence and mutated homology arms for Ccel_3258 (*ctaC*) disruption	This study
pCasC-*ctaC*	CRISPR/Cas vector for Ccel_3258 (*ctaC*) inactivation	[2]
pCTA1	Plasmid expressing Ccel_3262-Ccel_3257 under control of *lac* promoter	[2]
pCTA1-Δ*ctaC*	pCTA1-derivative lacking *ctaC* (Ccel_3258)	This study
pCTA2	Plasmid expressing Ccel_3256-Ccel_3250 under control of *trc* promoter	[2]
pET28a	Expression vector for N-terminally His_6-tagged proteins	Novagen
pET28-*ctaC*	N-terminal His_6-tag on Ccel_3258	This study
pET28-*ctaE*	N-terminal His_6-tag on Ccel_3256	This study
pET28-$ctaE_{S36A}$	S36A mutant of CtaE	This study
pET28-*ctaH*	N-terminal His_6-tag on Ccel_3253	This study
pET28-$ctaH_{S40A}$	S40A mutant of CtaH	This study
pMAL-C5E	Expression vector for N-terminally MBP-tagged proteins	NEB
pMAL-*ctaC*	N-terminal MBP-tag on Ccel_3258	This study
pMAL-$ctaC_{D77A}$	D77A mutant of CtaC	This study
pMAL-$ctaC_{S78A}$	S78A mutant of CtaC	This study
pMAL-$ctaC_{C154A}$	C154A mutant of CtaC	This study
pMAL-$ctaC_{C157A}$	C157A mutant of CtaC	This study
pMAL-$ctaC_{C284A}$	C284A mutant of CtaC	This study

Table S3. Oligonucleotide primers used in this study. Base pairs changed for gene mutagenesis are bolded and underlined.

**Name**	**Sequence**	**Use**
pMAL-Seq-F	GGTCGTCAGACTGTCGATGAAGCC	pMAL-C5E sequencing
pMAL-Seq-R	TGTCCTACTCAGGAGAGCGTTCAC	pMAL-C5E sequencing
T7 Seq F	TAATACGACTCACTATAGGG	pET28a sequencing
T7 Seq R	GCTAGTTATTGCTCAGCGG	pET28a sequencing
TargetCT-F	GCAATGATACCGCGAGACCATATGCTGGATCCTTGAC	pCasC-*ctaC* cloning
TargetCT-R	GGTTTCGGTCTCCACGCAGCTTGCATGTCTGCAGGCC	pCasC-*ctaC* cloning
pCasC-seq	AACAAGCCATGAAAACCG	pCasC-ctaC sequencing
Ccel_3258 F	GGTGCCGCGCGGCAGCCATATGATTGAATGTGAGGTAAAGTGT AAAAAG	pET28-*ctaC* cloning
Ccel_3258 R	GGTGCTCGAGTGCGGCCGCAAGCTTTCATTTTTCACCTACCCA AAATAG	pET28-*ctaC* cloning
Ccel_3258 F2	AAAGGTACCAATGATTGAATGTGAGGTAAAGTG	pMAL-*ctaC* cloning
Ccel_3258 R2	AAAGGATCCTCATTTTTCACCTACCCAAAATAG	pMAL-*ctaC* cloning
Ccel_3256 F	AAAGCTAGCATGAAAGAAAAAATCAAGGAGATTATATG	pET28-*ctaE* cloning
Ccel_3256 R	TTTGGATCCCTAAACAGATTTTACCGATACATATTTCC	pET28-*ctaE* cloning
Ccel_3253 F	AAAGCTAGCTTGAATTATTCTGACTATGATAAATTTC	pET28-*ctaH* cloning
Ccel_3253 R	TTTGGATCCTTATACTTCCAGCTCATTATATTGATTTG	pET28-*ctaH* cloning
CtaE S36A F	GGGATTAGAT**G**CCATAAATGTAGTTTATATTATTGG	SDM of pET28-*ctaE*
CtaE S36A R	CTACATTTATGG**C**ATCTAATCCCAATTCTGTAAG	SDM of pET28-*ctaE*
CtaH S40A F	ATTTTTAGAT**G**CAATCTCCTTCTATACCTTAATTG	SDM of pET28-*ctaH*
CtaH S40A R	AGAAGGAGATTG**C**ATCTAAAAATAAATCCTCTTTTAAATCG	SDM of pET28-*ctaH*
CtaC-D77A F	GGAAAAG**C**CAGTATATATGTTGCTTACATACTATCAAAAGTC	SDM of pMAL-*ctaC*
CtaC-D77A R	CATATATACTG**G**CTTTTCCCCCGCTTAAC	SDM of pMAL-*ctaC*
CtaC-S78A F	GAAAAGAC**GCA**ATATATGTTGCTTACATACTATCAAAAGTC	SDM of pMAL-*ctaC*
CtaC-S78A R	CATATAT**TGC**GTCTTTTCCCCCGCTTAACATTACC	SDM of pMAL-*ctaC*
CtaC-C154A F	CCAATTA**GC**TTTCTTCTGCGGGAGACTCC	SDM of pMAL-*ctaC*
CtaC-C154A R	AGAAGAAA**GC**TAATTGGCTGCAATCCTGCAAC	SDM of pMAL-*ctaC*
CtaC-C157A F	TTTCTTC**GC**CGGGAGACTCCTGAAAAC	SDM of pMAL-*ctaC*
CtaC-C157A R	GTCTCCCG**GC**GAAGAAACATAATTGGCTGCAATC	SDM of pMAL-*ctaC*
CtaC-C284A F	GTCAGGA**GCT**AAACTCGGCAAACTTATGG	SDM of pMAL-*ctaC*
CtaC-C284A R	CGAGTTTA**GC**TCCTGACGAGATATATTTCTTGG	SDM of pMAL-*ctaC*
pCTA1-Δ*ctaC* F	CATCCAAAGAGAAGCTATTTTGGG	Deletion of *ctaC* from pCTA1
pCTA1-Δ*ctaC* R	TTAGATACCACTACTATTGCTTATAGCTG	Deletion of *ctaC* from pCTA1
Δ*ctaC* screen F	AAAGCTAGCATGGGGAGCACATACTGGAG	PCR screen for pCTA1-Δ*ctaC*
Δ*ctaC* screen R	TTAATTGATTACTTTAATCAAATTATCCATTTCTTCATAACG	PCR screen for pCTA1-Δ*ctaC*
Cas-3258-check F	CGGAAAGCGACTTGGACAACATAGTC	Amplification of *ctaC* for CRISPR/Cas mutant verification
Cas-3258-check R	CTTCTTGTCTTCCCATTCATGCTCGATTCCTG	Amplification of *ctaC* for CRISPR/Cas mutant verification
Cas-3258-seq	GCGACTTGGACAACATAGTCAAAATAG	*ctaC* CRISPR/Cas mutant sequencing

Table S4. List of AANH proteins used to construct phylogenetic tree.

Accession number	Organism	Class
CtaC (Ccel_3258)	*Ruminiclostridium cellulolyticum*	CtaC
CCH48483.1	*Pseudodesulfovibrio piezophilus* C1TLV30	CtaC
WP_035914923.1	*Fusobacterium necrophorum*	CtaC
PYG86578.1	*Ruminiclostridium sufflavum* DSM 19573	CtaC
2DET	*Escherichia coli*	MnmA
CRQ40439.1	*Pseudomonas aeruginosa*	MnmA
WP_049318720.1	*Staphylococcus aureus*	MnmA
NP_010251.1	*Saccharomyces cerevisiae* S288C	MnmA
ACI80040.1	*Escherichia coli*	GMP Synthetase*
NP_499865.1	*Caenorhabditis elegans*	Ncs6
NP_177744.2	*Arabidopsis thaliana*	Ncs6
KZV11018.1	*Saccharomyces cerevisiae*	Ncs6
AEI34198.1	*Clostridium acetobutylicum*	ThiI
WP_011279120.1	*Sulfolobus acidocaldarius*	ThiI
2C5S	*Bacillus anthracis*	ThiI
4KR6	*Thermotoga maritima* MSB8	ThiI
CAQ30892.1	*Escherichia coli*	ThiI
WP_042395217.1	*Clostridium disporicum*	TtcA
Ccel_3405	*Ruminiclostridium cellulolyticum*	TtcA
EGT70038.1	*Escherichia coli*	TtcA
KWX42002.1	*Pseudomonas aeruginosa*	TtcA
5B4F	*Thermus thermophilus* HB27	TtuA
ADU52157.1	*Thermaerobacter marianensis* DSM 12885	TtuA
ADU96609.1	*Thermovibrio ammonificans* HB-1	TtuA
WP_051288339.1	*Photobacterium halotolerans*	YcfA
WP_034946289.1	*Erwinia oleae*	YcfA
YcfA (CAC05486.1)	*Erwinia amylovora*	YcfA

*Used as an outgroup to root the phylogenetic tree.

Table S5. List of AANH proteins used to construct sequence similarity network. Black, strict anaerobe; Red, facultative anaerobe; Blue, microaerophilic; Purple, strict aerobe.

Accession number	Organism	Cluster type	Phylum		Accession number	Organism	Cluster type	Phylum
WP_004616073.1	*Ruminiclostridium papyrosolvens*	NCTT	Firmicutes		WP_085280468.1	*Clostridium botulinum*	NRPS/PKS	Firmicutes
WP_020815968.1	*Ruminiclostridium papyrosolvens*	NCTT	Firmicutes		WP_061319462.1	*Clostridium botulinum*	NRPS/PKS	Firmicutes
WP_010244015.1	*Hungateiclostridium cellulolyticum*	NCTT	Firmicutes		WP_011986905.1	*Clostridium botulinum*	NRPS/PKS	Firmicutes
WP_022620697.1	*Clostridioides difficile*	NCTT	Firmicutes		WP_101515161.1	*Clostridium botulinum*	NRPS/PKS	Firmicutes
WP_032840631.1	*Fusobacterium* spp.	NCTT	Fusobacteriaceae		WP_024931341.1	*Clostridium botulinum*	NRPS/PKS	Firmicutes
WP_035914923.1	*Fusobacterium necrophorum*	NCTT	Fusobacteriaceae		WP_004441700.1	*Clostridium botulinum*	NRPS/PKS	Firmicutes
WP_119234807.1	*Ruminococcus* sp. AF41-9	NCTT	Firmicutes		WP_066477838.1	*Sphingomonas* spp.	NRPS/PKS	Proteobacteria
WP_053337822.1	*Clostridium botulinum*	NCTT	Firmicutes		WP_007782084.1	*Desulfosporosinus youngiae*	NRPS/PKS	Firmicutes
WP_015926605.1	*Ruminiclostridium cellulolyticum*	NCTT	Firmicutes		WP_085160327.1	*Alteromonadaceae* bacterium Bs31	NRPS/PKS	Proteobacteria
WP_036675459.1	*Paenibacillus* spp.	NRPS	Firmicutes		WP_021170794.1	*Sporomusa* spp.	NRPS/PKS	Firmicutes
WP_025848117.1	*Paenibacillus ehimensis*	NRPS	Firmicutes		WP_121467821.1	*Alteromonadaceae* bacterium 2052S.S.stab0a.01	NRPS/PKS	Proteobacteria
WP_010494743.1	*Paenibacillus elgii*	NRPS	Firmicutes		WP_044618538.1	*Gynuella sunshinyii*	NRPS/PKS	Proteobacteria
WP_063184537.1	*Paenibacillus elgii*	NRPS	Firmicutes		WP_110462907.1	*Ruminiclostridium sufflavum*	NRPS/PKS	Firmicutes
WP_108533645.1	*Paenibacillus elgii*	NRPS	Firmicutes		WP_095366310.1	*Pseudoalteromonas* sp. NBT06-2	NRPS/PKS	Proteobacteria
WP_090671113.1	*Paenibacillus tianmuensis*	NRPS	Firmicutes		WP_078684178.1	*Desulfovibrio bizertensis*	NRPS/PKS	Proteobacteria
WP_025693864.1	*Paenibacillus durus*	NRPS	Firmicutes		WP_075753435.1	*Sporomusa sphaeroides*	NRPS/PKS	Firmicutes
WP_025692841.1	*Paenibacillus zanthoxyli*	NRPS	Firmicutes		WP_011367558.1	*Desulfovibrio alaskensis*	NRPS/PKS	Proteobacteria
WP_042207049.1	*Paenibacillus durus*	NRPS	Firmicutes		WP_051363840.1	*Desulfovibrio alaskensis*	NRPS/PKS	Proteobacteria
WP_055038179.1	*Blastochloris viridis*	NRPS	Proteobacteria		WP_080066160.1	*Ruminiclostridium hungatei*	NRPS/PKS	Firmicutes
WP_029084249.1	*Bradyrhizobium* sp. th.b2	NRPS	Proteobacteria		WP_040758856.1	*Ruminiclostridium papyrosolvens*	NRPS/PKS	Firmicutes
WP_011472505.1	*Rhodopseudomonas palustris*	NRPS	Proteobacteria		WP_114296896.1	*Anaerobacterium chartisolvens*	NRPS/PKS	Firmicutes
WP_114296687.1	*Anaerobacterium chartisolvens*	NRPS	Firmicutes		WP_074112462.1	*Paenibacillus* sp. P46E	NRPS/PKS	Firmicutes
WP_024831254.1	*Ruminiclostridium josui*	NRPS	Firmicutes		WP_054750342.1	*Ruminiclostridium josui*	Other	Firmicutes
WP_029434396.1	*Bilophila wadsworthia*	NRPS	Proteobacteria		WP_006862528.1	*Marvinbryantia formatexigens*	Other	Firmicutes
WP_005026365.1	*Bilophila wadsworthia*	NRPS	Proteobacteria		WP_066060481.1	Candidatus *Desulfofervidus auxilii*	Other	Proteobacteria
WP_009378980.1	*Bilophila* sp. 4_1_30	NRPS	Proteobacteria		WP_016216509.1	*Eubacterium* sp. 14-2	Other	Firmicutes
WP_014959011.1	*Desulfobacula toluolica*	NRPS	Proteobacteria		WP_044943817.1	*Blautia schinkii*	Other	Firmicutes
WP_027361890.1	*Halodesulfovibrio aestuarii*	NRPS	Proteobacteria		WP_115482638.1	*Robinsoniella* sp. MCWD5	Other	Firmicutes
WP_019999576.1	*Desulfovibrionaceae*	NRPS	Proteobacteria		WP_069194972.1	*Clostridium* spp.	Other	Firmicutes
WP_049962566.1	*Ruminococcus* sp. HUN007	NRPS/PKS	Firmicutes		WP_084070044.1	*Desulfobacterium vacuolatum*	Other	Proteobacteria
WP_033065014.1	*Clostridium botulinum*	NRPS/PKS	Firmicutes		WP_054740290.1	*Cellulosilyticum ruminicola*	Other	Firmicutes
WP_015924503.1	*Ruminiclostridium cellulolyticum*	NRPS/PKS	Firmicutes		WP_024832090.1	*Ruminiclostridium josui*	Other	Firmicutes
WP_007957526.1	*Pelosinus fermentans*	NRPS/PKS	Firmicutes		WP_005997600.1	*Desulfuromonas acetoxidans*	Other	Proteobacteria
WP_090933233.1	*Pelosinus propionicus*	NRPS/PKS	Firmicutes		WP_074929510.1	*Nitrosomonas eutropha*	Other	Proteobacteria
WP_007930405.1	*Pelosinus* spp.	NRPS/PKS	Firmicutes		WP_011112727.1	*Nitrosomonas europaea*	Other	Proteobacteria
WP_035780772.1	*Butyrivibrio* sp. LB2008	NRPS/PKS	Firmicutes		WP_090332872.1	*Nitrosomonas* sp. Nm51	Other	Proteobacteria
WP_050198440.1	*Streptococcus agalactiae*	NRPS/PKS	Firmicutes		WP_087439712.1	*Sulfurospirillum* spp.	Other	Proteobacteria
WP_004440624.1	*Clostridium botulinum*	NRPS/PKS	Firmicutes		WP_014259521.1	*Desulfovibrio africanus*	Other	Proteobacteria

Accession number	Organism	Cluster type	Phylum		Accession number	Organism	Cluster type	Phylum
WP_076235220.1	*Clostridium botulinum*	NRPS/PKS	Firmicutes		WP_005989424.1	*Desulfovibrio africanus*	Other	Proteobacteria
WP_045540449.1	*Clostridium botulinum*	NRPS/PKS	Firmicutes		WP_085052012.1	*Nitrospirae* bacterium HCH-1	Other	Proteobacteria
WP_003403968.1	*Clostridium botulinum*	NRPS/PKS	Firmicutes		WP_112939578.1	*Massilia* sp. YMA4	Other	Proteobacteria
WP_085296116.1	*Clostridium botulinum*	NRPS/PKS	Firmicutes		WP_107139650.1	*Massilia armeniaca*	Other	Proteobacteria
WP_003484596.1	*Clostridium* spp.	NRPS/PKS	Firmicutes		WP_082616321.1	*Massilia* sp. Root418	Other	Proteobacteria
WP_045516152.1	*Clostridium sporogenes*	NRPS/PKS	Firmicutes		WP_107869419.1	*Massilia* spp.	Other	Proteobacteria
WP_061294425.1	*Clostridium botulinum*	NRPS/PKS	Firmicutes		WP_014468375.1	*Arcobacter butzleri*	Other	Proteobacteria
WP_030035820.1	*Clostridium botulinum*	NRPS/PKS	Firmicutes		WP_081456264.1	*Candidatus Pelagibacter* sp. IMCC9063	Other	Proteobacteria
WP_076178411.1	*Clostridium botulinum*	NRPS/PKS	Firmicutes		WP_006583742.1	*Thermanaerovibrio velox*	Other	Synergistetes
WP_096043484.1	*Clostridium botulinum*	NRPS/PKS	Firmicutes		WP_012869425.1	*Thermanaerovibrio acidaminovorans*	Other	Firmicutes
WP_012343961.1	*Clostridium botulinum*	NRPS/PKS	Firmicutes		WP_109324552.1	*Pseudarcicella* sp. HME7025	Other	Baceroidetes
WP_099839557.1	*Clostridium combesii*	NRPS/PKS	Firmicutes		WP_066619036.1	*Clostridium magnum*	Other	Firmicutes

References

[1] T. Lincke, S. Behnken, K. Ishida, M. Roth, C. Hertweck, *Angew. Chem. Int. Ed.* **2010**, *49*, 2011-2013.

[2] K. L. Dunbar, H. Buttner, E. M. Molloy, M. Dell, J. Kumpfmuller, C. Hertweck, *Angew. Chem. Int. Ed.* **2018**, *57*, 14080-14084.

[3] K. Katoh, J. Rozewicki, K. D. Yamada, *Brief Bioinform* **2017**.

[4] K. Tamura, G. Stecher, D. Peterson, A. Filipski, S. Kumar, *Mol. Biol. Evol.* **2013**, *30*, 2725-2729.

[5] F. Sievers, A. Wilm, D. Dineen, T. J. Gibson, K. Karplus, W. Li, R. Lopez, H. McWilliam, M. Remmert, J. Soding, J. D. Thompson, D. G. Higgins, *Mol. Syst. Biol.* **2011**, *7*, 539.

[6] J. A. Gerlt, J. T. Bouvier, D. B. Davidson, H. J. Imker, B. Sadkhin, D. R. Slater, K. L. Whalen, *Biochim. Biophys. Acta* **2015**, *1854*, 1019-1037.

[7] P. Shannon, A. Markiel, O. Ozier, N. S. Baliga, J. T. Wang, D. Ramage, N. Amin, B. Schwikowski, T. Ideker, *Genome Res* **2003**, *13*, 2498-2504.

[8] F. Kloss, T. Lincke, C. Hertweck, *Eur. J. Org. Chem.* **2011**, *2011*, 1429-1431.

[9] F. Kloss, A. I. Chiriac, C. Hertweck, *Chem. Eur. J.* **2014**, *20*, 15451-15458.

[10] K. Blin, L. E. Pedersen, T. Weber, S. Y. Lee, *Synth. Syst. Biotechnol.* **2016**, *1*, 118-121.

[11] L. Fluhe, O. Burghaus, B. M. Wieckowski, T. W. Giessen, U. Linne, M. A. Marahiel, *J. Am. Chem. Soc.* **2013**, *135*, 959-962.

[12] H. Beinert, *Anal. Biochem.* **1983**, *131*, 373-378.

[13] A. Litomska, K. Ishida, K. L. Dunbar, M. Boettger, S. Coyne, C. Hertweck, *Angew. Chem. Int. Ed.* **2018**, *57*, 11574-11578.

[14] S. Behnken, T. Lincke, F. Kloss, K. Ishida, C. Hertweck, *Angew. Chem. Int. Ed.* **2012**, *51*, 2425-2428.

[15] S. Gruenewald, H. D. Mootz, P. Stehmeier, T. Stachelhaus, *Appl. Environ. Microbiol.* **2004**, *70*, 3282-3291.
